# Phenotypic and Genomic Characterization of *Bacillus* sp. DLB3, a Black Pepper Rhizosphere Isolate with Antagonistic, Abiotic Stress-Tolerant, and Antibiotic-Susceptible Traits

**DOI:** 10.3390/ijms27219536

**Published:** 2026-10-25

**Authors:** Anh Thi Lan Vu, Son Nghia Hoang, Chi Nguyen Quynh Ho, Cuong Phan Minh Le, Huy Nghia Quang Hoang, Han Thai Minh Nguyen, Long Thanh Le

**Affiliations:** 1 Institute of Life Sciences, Vietnam Academy of Science and Technology, Ho Chi Minh City 70000, Vietnam; vtlanh@ils.vast.vn (A.T.L.V.); hoangnghiason123@gmail.com (S.N.H.); hnqchi@ils.vast.vn (C.N.Q.H.); lpmcuong@ils.vast.vn (C.P.M.L.); hnqhuy@ils.vast.vn (H.N.Q.H.); 2 Graduate University of Science and Technology, Vietnam Academy of Science and Technology, Hanoi 100000, Vietnam; 3 Biotechnology Innovation Center, University of New Hampshire, Manchester, NH 03101, USA; han.nguyen@unh.edu

**Keywords:** *Bacillus velezensis*, biocontrol, heavy metal tolerance, genome mining, biosynthetic gene cluster, antibiotic resistance gene

## Abstract

Rhizosphere bacteria combining biocontrol activity with resilience to environmental stress are valuable candidates for sustainable agriculture. This study characterized strain DLB3, isolated from black pepper rhizosphere soil, through dual-culture and disk-diffusion antagonism assays, enzymatic and siderophore screening, physiological tolerance tests, antibiotic susceptibility testing, whole-genome sequencing, and genome mining. Phylogenomic analysis and average nucleotide identity (>97%) and digital DNA–DNA hybridization (>79%) values identified the isolate as *Bacillus velezensis*. The strain inhibited *Fusarium falciforme*, *Fusarium oxysporum*, and *Colletotrichum gloeosporioides* (47.6–71.2% radial growth inhibition) and produced an inhibition zone (8.4 mm) against *Ralstonia solanacearum*, alongside extracellular protease, amylase, CMCase, and catecholate-type siderophore production. DLB3 tolerated a broad range of temperatures (20–50 °C, with optimal growth at 25–40 °C, peaking at 35 °C), pH (5–9), and salinity (up to 10% NaCl), and exhibited minimum inhibitory concentrations (MICs) of 1800, 500, 450, and 250 mg L^−1^ for Pb, Zn, Cu, and Cr, respectively, while remaining susceptible to all nine antibiotics tested. The 3,913,361 bp draft genome was assembled into 44 contigs (N50 = 990,239 bp; L50 = 2), with 99.81% completeness and 0.12% contamination according to CheckM. It harbored 18 biosynthetic gene clusters, carbohydrate-active enzymes, peptidases, and genes associated with heavy metal resistance, broadly consistent with the observed phenotypes, as well as a homolog of the cfr methyltransferase gene (97.4% identity) that was not phenotypically expressed. These findings provide a comprehensive genomic and phenotypic profile of strain DLB3 and its biocontrol-related potential.

## 1. Introduction

Meeting the growing demand for food represents a major challenge for global agriculture, with the world population expected to approach 9.7 billion by 2050 [1]. Currently, plant pathogens and pests are a major cause of crop yield loss, accounting for an estimated 20–40% reduction in global agricultural production and directly affecting the global economy and food security [2,3]. While synthetic fertilizers and chemical pesticides have been essential for maximizing crop yields, their excessive and improper use has led to serious concerns regarding soil degradation, environmental contamination, and risks to human health [2,4]. In this context, plant growth-promoting rhizobacteria (PGPR), such as species from the genera *Bacillus*, *Pseudomonas*, *Rhizobium*, and *Agrobacterium*, are recognized as sustainable and eco-friendly alternatives to traditional agrochemicals [5,6,7].

*Bacillus velezensis* is a Gram-positive, rod-shaped, aerobic, endospore-forming bacterium and a member of the *Bacillus subtilis* species complex, widely studied as a PGPR in sustainable agriculture [8,9]. It acts as an effective biocontrol agent by producing diverse antimicrobial metabolites, including lipopeptides (surfactin, iturin, and fengycin) and polyketides (macrolactin, bacillaene, and difficidin), as well as the secretion of hydrolytic enzymes that can degrade pathogen cell walls and the induction of systemic resistance in host plants [10,11,12]. Beyond biocontrol, several *B. velezensis* strains have also been reported to promote plant growth through phytohormone production and nutrient mobilization [13,14], highlighting the diverse beneficial traits associated with this species. Recent advances in whole-genome sequencing and genome mining have provided critical insights into these beneficial capabilities by enabling the identification of key biosynthetic gene clusters (BGCs) and functional gene repertoires underlying antimicrobial activity, while also providing molecular evidence for accurate taxonomic classification within the closely related *B. subtilis* complex [2,8,15].

In addition to biotic stress imposed by phytopathogens, rhizobacteria in agricultural soils encounter a range of abiotic stresses, including variations in pH and temperature, salinity, and exposure to heavy metals, which can influence their survival and persistence in the rhizosphere [16,17]. The ability to tolerate these environmental stresses while maintaining plant-beneficial and biocontrol traits may be important for the successful establishment and activity of rhizobacteria under variable soil conditions [16,17,18]. Therefore, characterization of both biotic interactions and abiotic stress tolerance is relevant for assessing the ecological and agricultural potential of rhizosphere-associated bacteria. In parallel, the use of live bacterial inoculants in agriculture highlights the importance of evaluating antibiotic susceptibility and resistance-associated genes in candidate PGPR strains [19]. Combining phenotypic susceptibility testing with genomic analysis can provide a more comprehensive assessment of their antibiotic resistance profiles and support their safety evaluation for agricultural use [19].

Although previous studies have combined phenotypic and genomic characterization of *B. velezensis* strains, the extent of integration across biocontrol activity, stress tolerance, and antibiotic susceptibility varies considerably among strains. In this study, we characterized *Bacillus* sp. DLB3, a strain previously isolated from black pepper rhizosphere soil, using an integrated genomic and phenotypic approach. The objectives were to (i) evaluate its antagonistic activity against fungal and bacterial phytopathogens; (ii) assess its physiological tolerance to temperature, pH, salinity, and heavy metals; (iii) determine its antibiotic susceptibility profile; and (iv) characterize its genome to identify genetic determinants potentially associated with these phenotypic traits.

## 2. Results

### 2.1. Morphological Characteristics of Bacillus sp. DLB3

**Figure 1:**
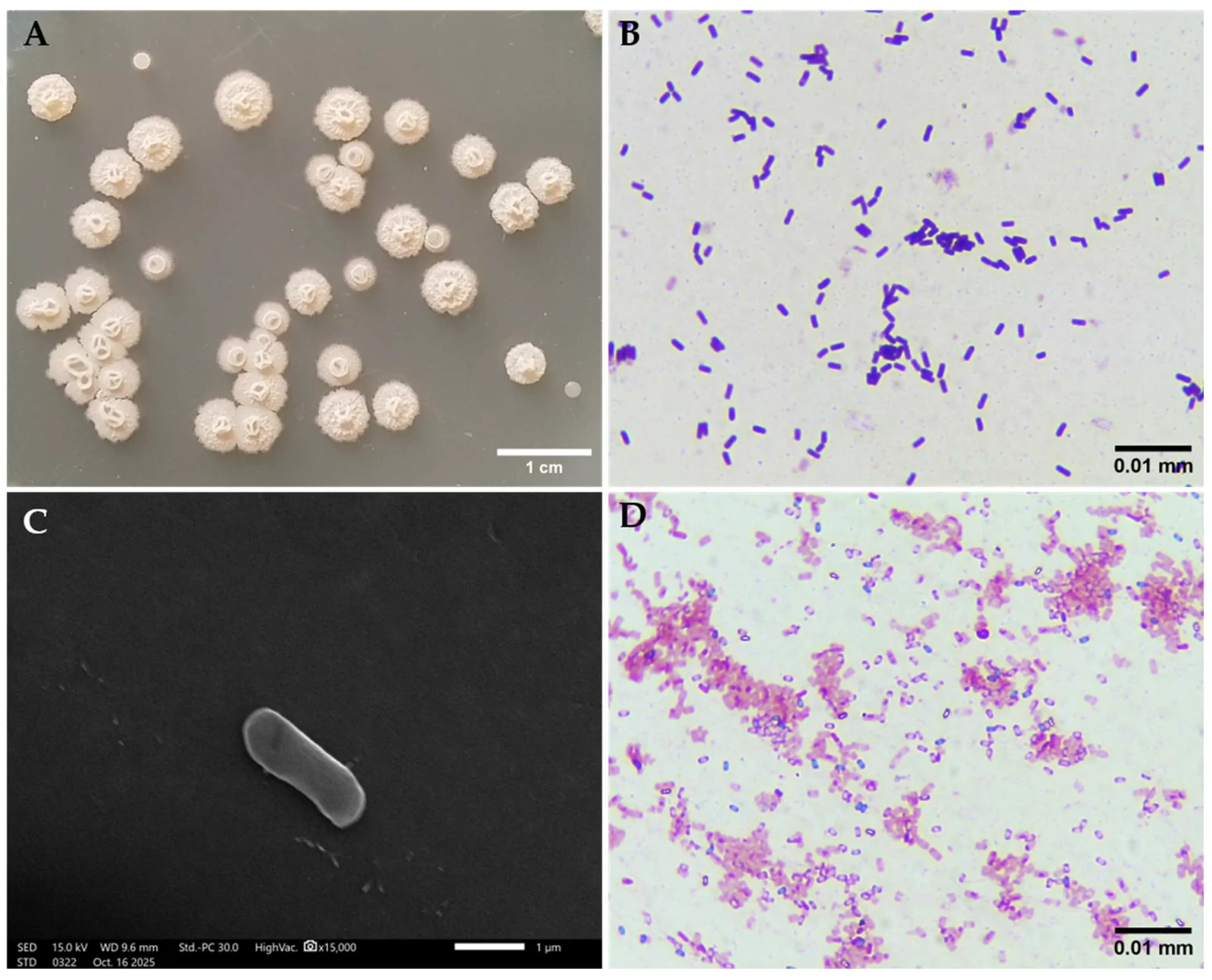
Morphological characteristics of strain DLB3. (**A**) Colony morphology on LB agar. (**B**) Gram-stained cells. (**C**) Scanning electron micrograph of DLB3 cell. (**D**) Endospore staining using the Schaeffer–Fulton method.

After incubation on LB agar at 37 °C for 24 h, colonies of strain DLB3 were creamy white, 1.7–7.0 mm in diameter, raised, with irregular margins and a dry, wrinkled surface (Figure 1A). Gram staining revealed Gram-positive, rod-shaped cells (Figure 1B). Scanning electron microscopy further confirmed the rod-shaped morphology, with cells measuring approximately 1.5–2.7 µm in length and 0.61–0.92 µm in width (Figure 1C). Endospore staining using the Schaeffer–Fulton method revealed oval endospores within the vegetative cells (Figure 1D), confirming that strain DLB3 is a Gram-positive, spore-forming bacterium consistent with the genus *Bacillus*.

### 2.2. Genome Assembly and Functional Annotation

**Figure 2:**
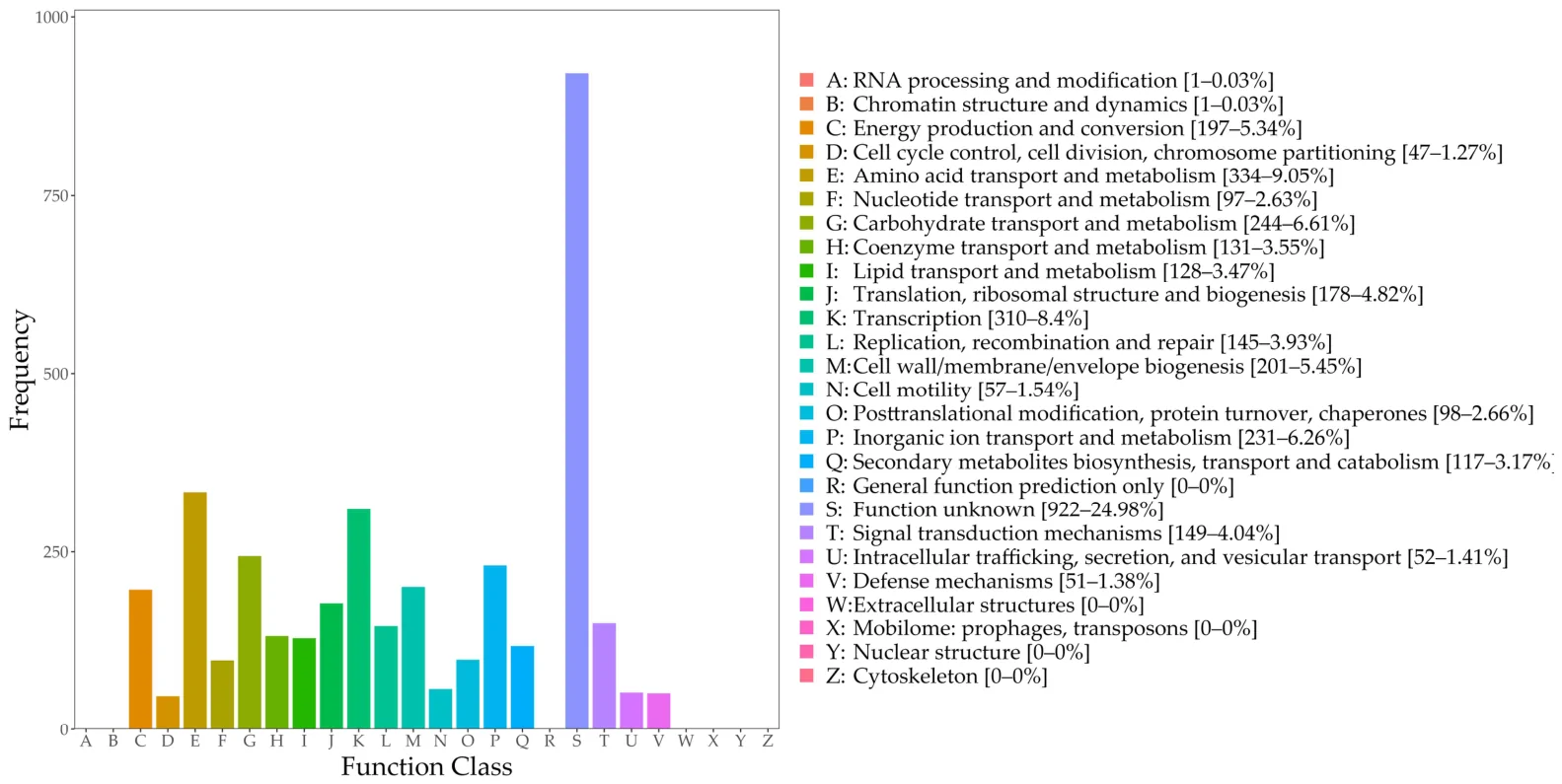
COG functional classification of predicted genes in the genome of strain DLB3.

Genome assembly of strain DLB3 yielded a total length of 3,913,361 bp across 44 contigs, with a G+C content of 46.45%, a largest contig of 1,115,001 bp, an N50 value of 990,239 bp, and an L50 of 2 (Table 1), indicating a relatively contiguous assembly. CheckM estimated genome completeness at 99.81% and contamination at 0.12%, with 0.00% strain heterogeneity, indicating a highly complete genome assembly with minimal contamination. The genome contained 3818 predicted genes, including 3740 coding sequences (CDSs), 74 tRNA genes, and 3 rRNA genes (Table 1). COG annotation assigned the predicted genes to multiple functional categories, with the most abundant groups being amino acid transport and metabolism (9.05%), transcription (8.40%), carbohydrate transport and metabolism (6.61%), and inorganic ion transport and metabolism (6.26%) (Figure 2). Notably, 24.98% of genes were classified as function unknown.

### 2.3. Phylogenomic and Taxonomic Classification of Strain DLB3

**Figure 3:**
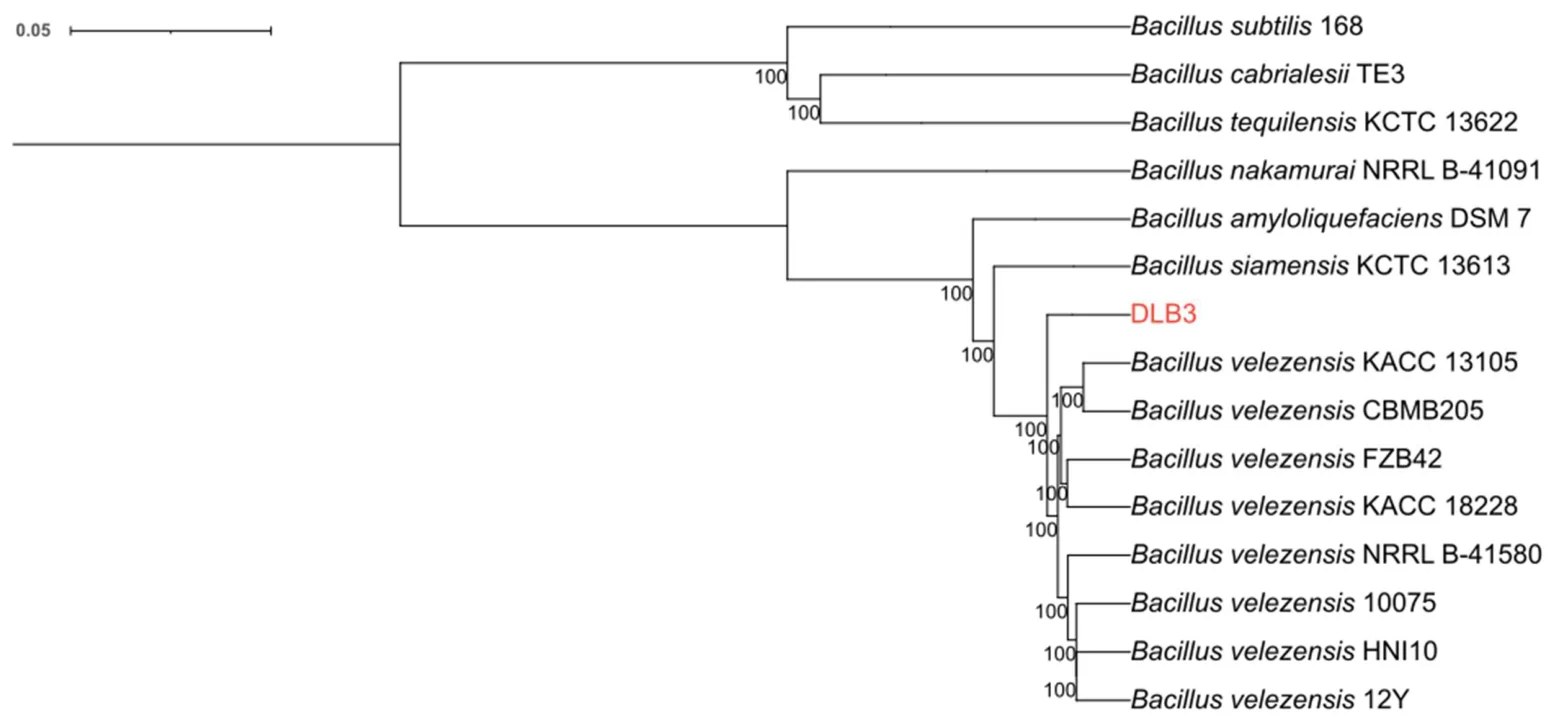
Core-genome phylogenetic tree of strain DLB3 and 14 reference *Bacillus* strains.

Pan-genome analysis performed using Roary identified a total of 17,322 gene clusters, of which 329 were defined as core genes shared by 99–100% of the analyzed genomes. The concatenated alignment of these core genes was used for phylogenomic reconstruction. The maximum-likelihood tree revealed that strain DLB3 clustered within the *B. velezensis* clade with strong bootstrap support (100%), clearly separated from the closely related species *B. amyloliquefaciens*, *B. siamensis*, *B. nakamurai*, *B. subtilis*, *B. cabrialesii*, and *B. tequilensis* (Figure 3).

**Figure 4:**
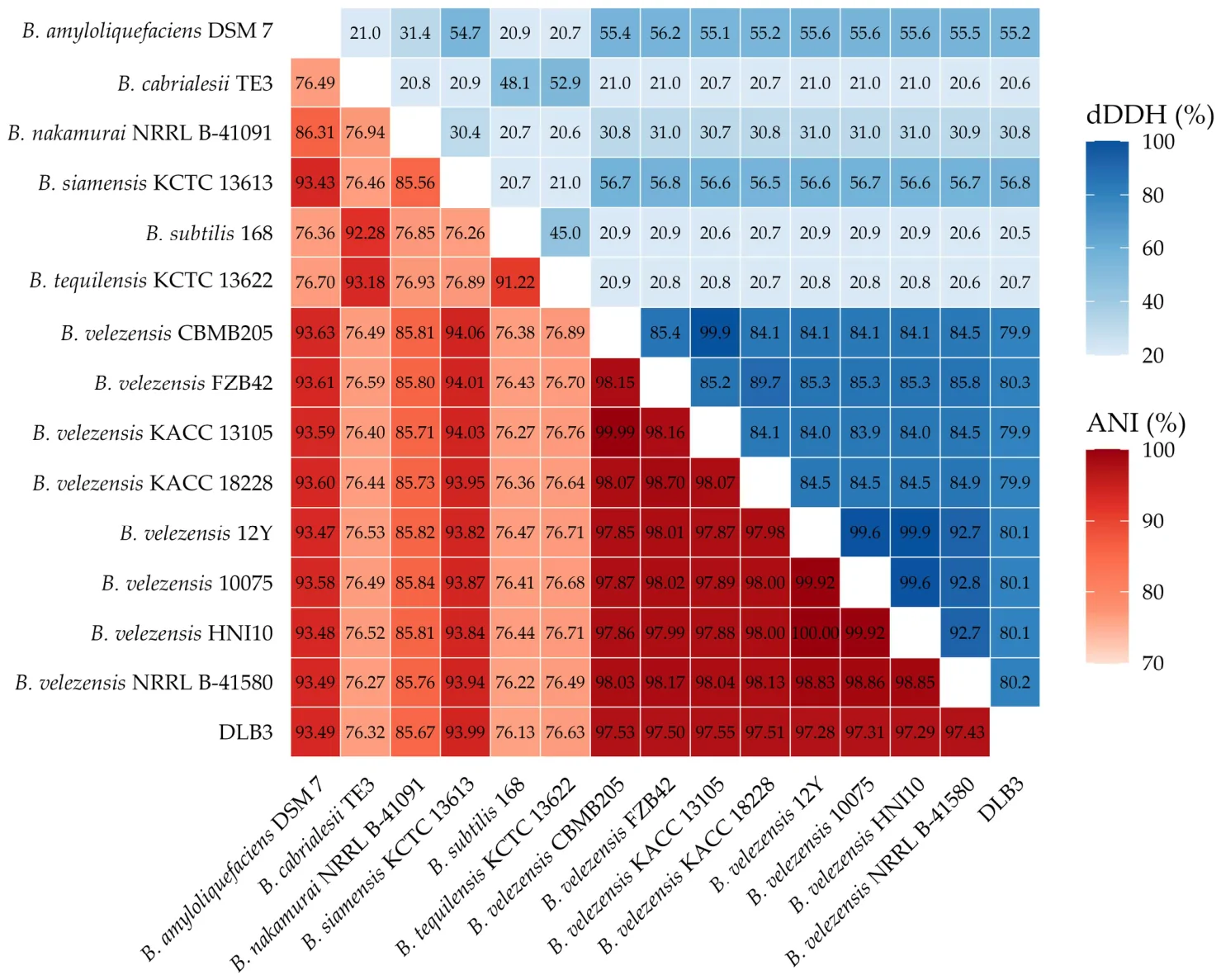
Heatmap of average nucleotide identity (ANI) and digital DNA–DNA hybridization (dDDH) values among strain DLB3 and 14 related *Bacillus* strains.

Average nucleotide identity (ANI) and digital DNA–DNA hybridization (dDDH) values were calculated between the genome of strain DLB3 and those of the 14 reference genomes (Figure 4). Strain DLB3 shared high ANI (>97%) and dDDH (>79%) values with all eight *B. velezensis* strains, including the type strain NRRL B-41580 (ANI = 97.43%, dDDH = 80.2%). Values were markedly lower with other reference species, particularly *B. subtilis* 168 and *B. cabrialesii* TE3 (ANI = 76.13–76.32%, dDDH = 20.5–20.6%), while intermediate ANI and dDDH values were observed with the phylogenetically closer relatives *B. amyloliquefaciens* DSM 7 (ANI = 93.49%, dDDH = 55.2%) and *B. siamensis* KCTC 13613 (ANI = 93.99%, dDDH = 56.8%). The highest ANI (97.55%) was obtained with *B. velezensis* KACC 13105, while the highest dDDH (80.3%) was observed with *B. velezensis* FZB42. Collectively, the phylogenomic tree together with the ANI and dDDH values, both exceeding the recognized species delineation thresholds (ANI ≥ 95–96%, dDDH ≥ 70%) [20,21,22], confirmed that strain DLB3 belongs to the species *B. velezensis*.

### 2.4. Antagonistic Activity Against Fungal and Bacterial Pathogens

**Figure 5:**
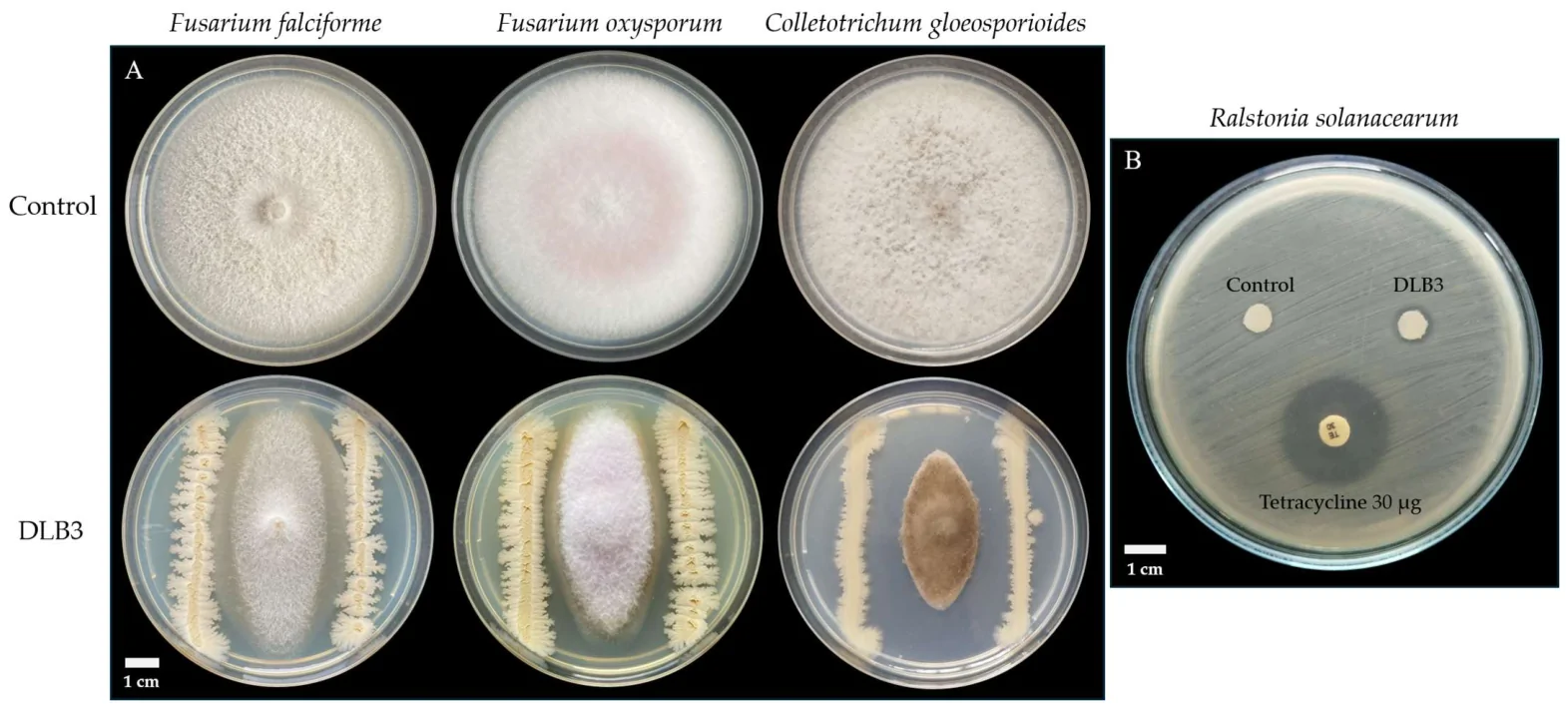
Antagonistic activity of strain DLB3 against fungal and bacterial phytopathogens. (**A**) Dual-culture assay showing the growth of *F. falciforme*, *F. oxysporum*, and *C. gloeosporioides* in the absence (Control) and presence (DLB3) of strain DLB3. (**B**) Disc diffusion assay against *R. solanacearum*, showing the cell-free supernatant of strain DLB3, sterile LB broth (Control), and tetracycline 30 μg.

To assess the antimicrobial activity of strain DLB3, assays were performed against four phytopathogens, including *F. falciforme*, *F. oxysporum*, *C. gloeosporioides*, and *R. solanacearum*. The results revealed that *B. velezensis* DLB3 exhibited antagonistic effects against all the tested pathogens. In the dual-culture assay, the percentage inhibition of radial growth was 47.6%, 54.1%, and 71.2% against *F. falciforme*, *F. oxysporum*, and *C. gloeosporioides*, respectively, with the highest inhibition observed for *C. gloeosporioides* (ANOVA, Tukey HSD, *p* < 0.001) (Figure 5A). Against the bacterial pathogen *R. solanacearum*, DLB3 produced an inhibition zone of 8.4 ± 0.1 mm (Figure 5B).

### 2.5. Production of Lytic Enzymes and Catecholate-Type Siderophore

**Figure 6:**
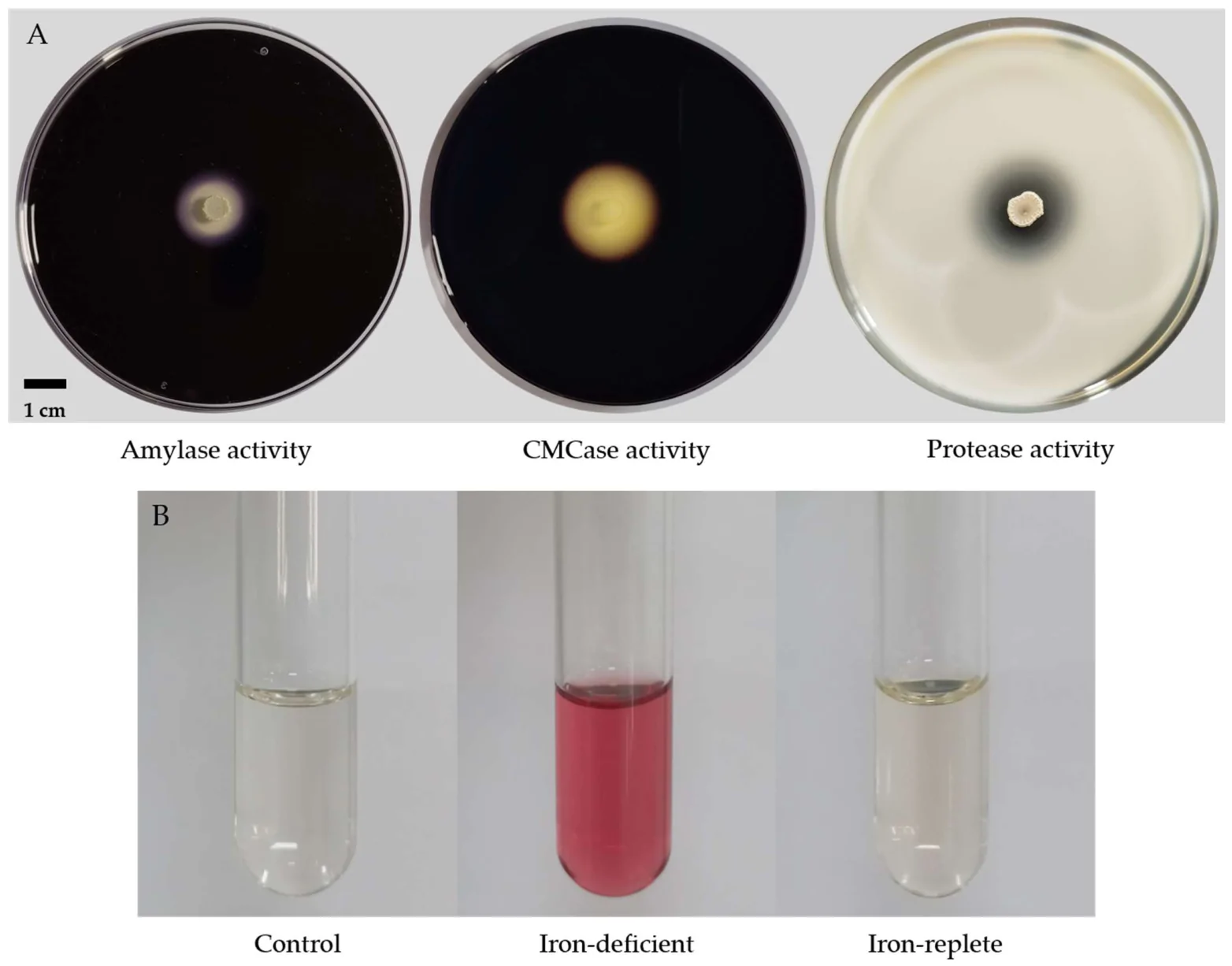
Extracellular hydrolytic enzyme activity and catecholate-type siderophore production by strain DLB3. (**A**) Hydrolysis zones on starch agar (amylase), CMC agar (CMCase), and casein agar (protease). (**B**) Color reaction of the Arnow assay under control (uninoculated), iron-deficient, and iron-replete conditions.

Strain DLB3 exhibited extracellular hydrolytic activity on all three substrate-specific media, producing clear hydrolysis zones with total diameters of 17.7 ± 0.5 mm on starch agar (amylase), 21.2 ± 0.6 mm on CMC agar (CMCase), and 15.1 ± 0.4 mm on casein agar (protease) (Figure 6A). Catecholate-type siderophore production was assessed using the Arnow assay. A marked color change from pale yellow to intense orange-red was observed in the culture filtrate of strain DLB3 grown under iron-deficient conditions (OD_510_ = 0.966 ± 0.062), compared with the uninoculated control (OD_510_ = 0.015 ± 0.001) (Figure 6B). Siderophore production was strongly suppressed under iron-replete conditions supplemented with FeCl_3_ (OD_510_ = 0.039 ± 0.003). These differences were significant (one-way ANOVA, F = 693.2, *p* < 0.0001), with Tukey’s HSD confirming higher values under iron deficiency than under both other conditions (*p* < 0.0001), whereas the control and iron-replete conditions did not differ significantly (*p* = 0.6968).

### 2.6. Growth Tolerance Under Stress Conditions

**Figure 7:**
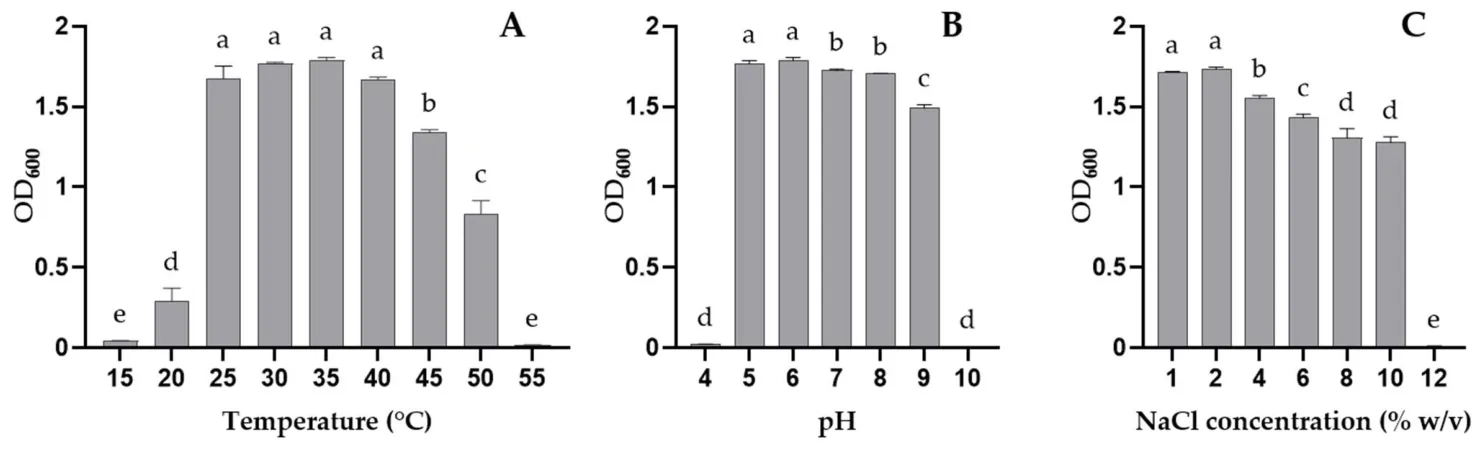
Growth of strain DLB3 under different (**A**) temperatures, (**B**) pH values, and (**C**) NaCl concentrations. Bars represent mean OD_600_ ± SD (*n* = 3); different letters above bars indicate significant differences (*p* < 0.05, Tukey’s HSD test).

Strain DLB3 displayed a broad range of growth tolerance to temperature, pH, and salinity (Figure 7A–C). Growth was optimal between 25 and 40 °C, with no significant differences observed within this range (*p* > 0.05), peaking at 35 °C (OD_600_ = 1.79 ± 0.02), which then declined sharply above 45 °C and was abolished at 55 °C. At lower temperatures, growth was markedly reduced at 20 °C and negligible at 15 °C. Similarly, optimal growth occurred at pH 5–6, with a gradual decline at higher pH values and no growth observed at pH 10. Strain DLB3 tolerated NaCl concentrations up to 10%, with the highest growth recorded at 1–2% NaCl, a progressive decline at higher salinity, and complete growth inhibition at 12% NaCl. Strain DLB3 also exhibited considerable tolerance to heavy metals, with MIC values of 1800, 500, 450, and 250 mg L^−1^ for Pb, Zn, Cu, and Cr, respectively, following the order Pb > Zn > Cu > Cr.

### 2.7. Antimicrobial Susceptibility Profile

The antimicrobial susceptibility of strain DLB3 was evaluated against nine antibiotics representing five classes, following EUCAST clinical breakpoints for *Bacillus* spp. Strain DLB3 was susceptible (S) to meropenem, imipenem, erythromycin, clindamycin, linezolid, and vancomycin, with inhibition zone diameters ranging from 17.5 to 38.2 mm (Table 2). For the fluoroquinolones, DLB3 produced inhibition zones of 23.7 mm for ciprofloxacin and 27.6 mm for levofloxacin. The norfloxacin screening test yielded an inhibition zone of 25.3 mm, exceeding the EUCAST screening threshold of 21 mm and was therefore interpreted as screen-negative. Accordingly, DLB3 was categorized as susceptible, with increased exposure (I) to both ciprofloxacin and levofloxacin according to the EUCAST criteria. No zone of inhibition was observed for the negative control disk. Overall, strain DLB3 showed susceptibility to all antibiotics tested, with no resistance detected among the nine agents evaluated.

### 2.8. Genome Mining Reveals Genetic Determinants Underlying Observed Phenotypes

Genome mining using antiSMASH (v8.0.4) predicted 18 biosynthetic gene clusters (BGCs), eight of which showed high similarity to known clusters in the MIBiG database, including fengycin, bacillaene, macrolactin H, bacilysin, bacillibactin, bacillothiazol A-N, surfactin, and andalusicin A/B (Table 3). These lipopeptide- and polyketide-associated clusters are consistent with the antagonistic activity of strain DLB3 observed against fungal and bacterial phytopathogens (Section 2.4), while the bacillibactin cluster corresponds to the catecholate-type siderophore activity detected in this study (Section 2.5). Additionally, several predicted clusters showed low or no similarity to known BGCs, suggesting the presence of potentially novel secondary metabolite biosynthetic pathways.

**Figure 8:**
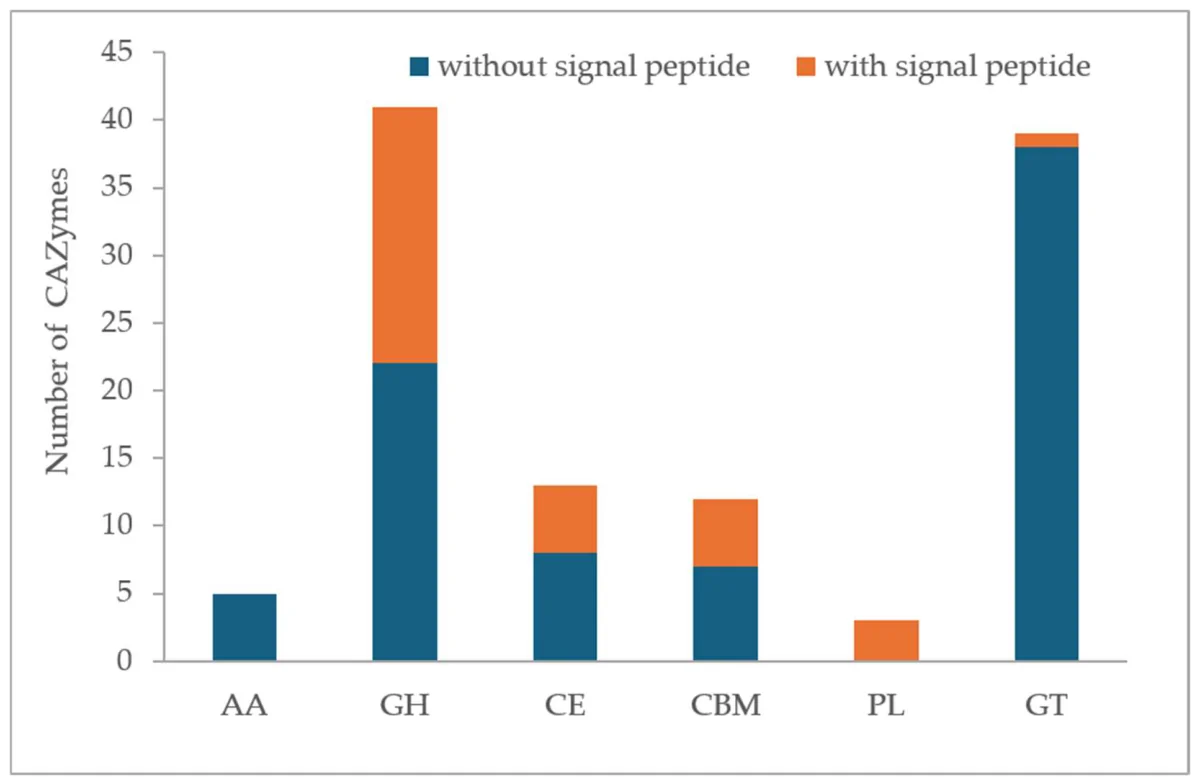
Classification of CAZymes in the genome of strain DLB3.

CAZyme annotation of the DLB3 genome, performed using dbCAN3 meta server and retaining only genes supported by at least two of the three integrated tools (HMMER, DIAMOND, dbCAN-sub), identified 113 genes distributed across six enzyme classes: 41 glycoside hydrolases (GHs), 39 glycosyltransferases (GTs), 13 carbohydrate esterases (CEs), 12 carbohydrate-binding modules (CBMs), 5 auxiliary activity enzymes (AAs), and 3 polysaccharide lyases (PLs) (Figure 8). Among these, 33 genes (29.2%) were predicted to contain signal peptides, indicating potential extracellular secretion. Within the GH class, one gene assigned to family GH13_28 (EC 3.2.1.1) was predicted to encode an α-amylase, consistent with the amylase activity detected on starch agar (Section 2.4). One gene (family GH5_2, EC 3.2.1.4) was putatively associated with the CMCase activity observed on CMC agar (Section 2.4). Additional GH families potentially relevant to fungal cell wall degradation were identified, including chitinases (GH18), a GH16 enzyme with predicted β-glucanase- and xylanase-related activities, a chitosanase-related family (GH46), and peptidoglycan-active lysozyme-like enzymes (GH23/GH73), which may contribute indirectly to the antagonistic activity of strain DLB3. BLASTp screening against the MEROPS database identified 73 candidate peptidase-encoding loci (Supplementary Table S1), including the extracellular proteases bacillolysin (95.7% identity) and subtilisin BPN’ (97.8% identity), consistent with the proteolytic activity observed on casein agar (Section 2.4).

Screening against the BacMet database identified 68 putative genes associated with metal resistance and homeostasis in strain DLB3 (Supplementary Table S2). Among the four heavy metals experimentally evaluated in this study, copper-associated genes were the most abundant, with 13 genes identified and sequence identities of up to 96.5%, including *yfmO*, *yfmP*, *copZ*, and *cueR*. Zinc-associated genes were also detected, comprising 13 genes with sequence identities of up to 51.8%, whereas five chromium-associated genes showed sequence identities of up to 61.7%. Notably, a single putative lead-resistance gene, *nmtR*, was identified, showing 45.5% sequence identity to the corresponding BacMet reference protein. Additional genes associated with untested metals (nickel, cadmium, mercury, manganese, and others) were identified, reflecting a broad genomic capacity for metal homeostasis.

Screening against the CARD database identified 10 antibiotic resistance genes under the “Strict” criterion (Supplementary Table S3). Most corresponded to antibiotic classes outside the tested panel, including tetracycline (*tet(45)*), fosfomycin (*FosBx1*), and cephalosporin (*BcI*) inactivation, as well as an efflux pump gene conferring resistance to the disinfecting agent benzalkonium chloride (*qacJ*, three loci). Three low-identity matches (33.7–34.9% identity) to the vancomycin resistance genes *vanT* (two loci) and *vanY* (one locus) were detected. However, no cognate vancomycin resistance ligase or *vanR*/*vanS* two-component regulatory system was identified elsewhere in the genome. Notably, a high-identity homolog (97.4%) of the *cfr* 23S rRNA methyltransferase gene, annotated as *clbA*, was also detected, despite strain DLB3 remaining phenotypically susceptible to clindamycin and linezolid.

### 2.9. Comparative Genomic Analysis

**Figure 9:**
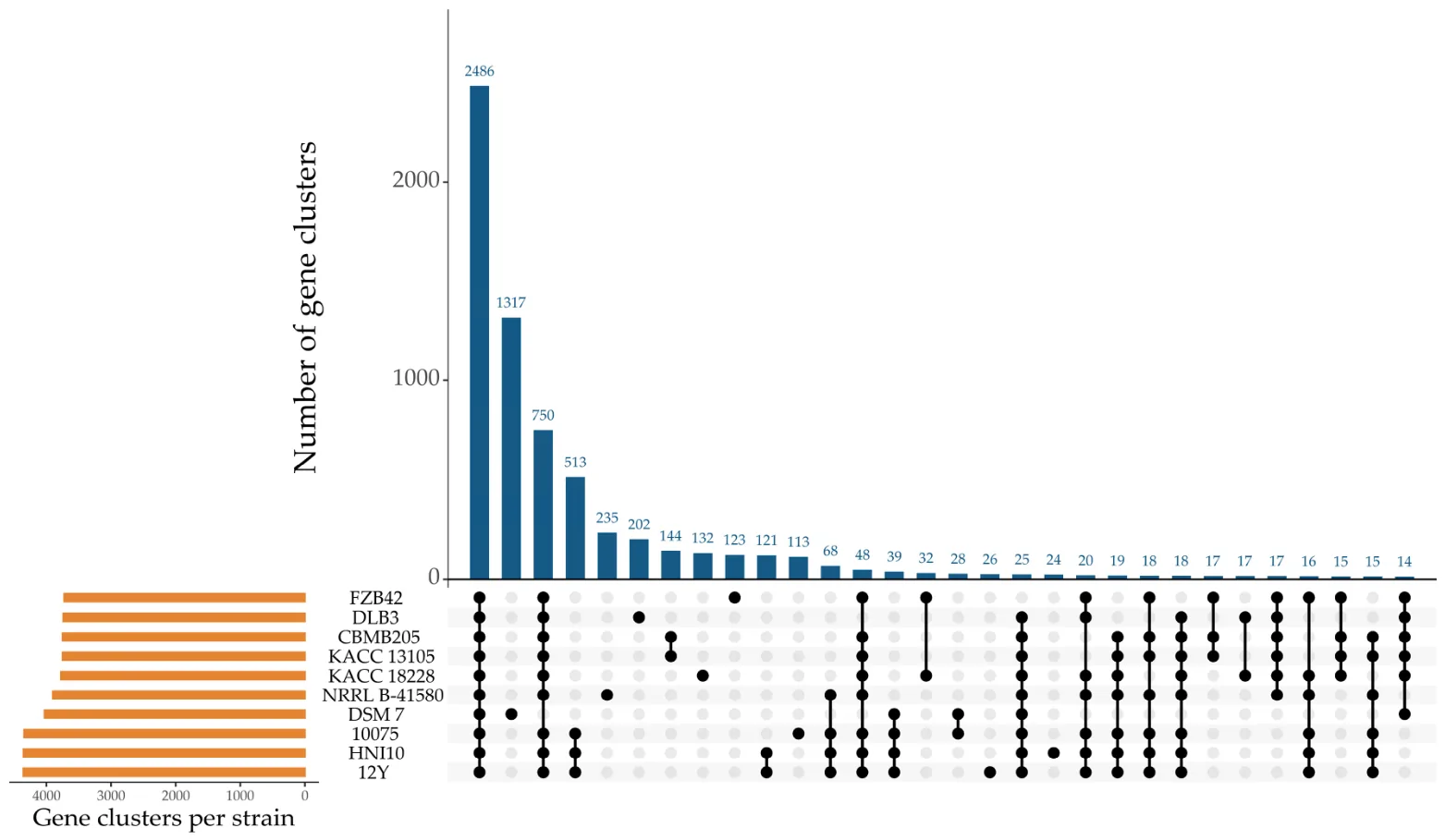
Distribution of gene clusters in the pan-genome of strain DLB3 and nine related strains. The horizontal bars represent the total number of gene clusters in each strain, while the vertical bars indicate the number of gene clusters in each intersection. Black dots and connecting lines represent the combinations of strains sharing the corresponding gene clusters.

Pan-genome analysis of strain DLB3 and nine reference genomes, comprising eight *B. velezensis* strains and *B. amyloliquefaciens* DSM 7, identified 6959 gene clusters, including 2486 core clusters (present in ≥99% of strains), 2294 shell clusters (present in 15–95% of strains), and 2179 cloud clusters (present in <15% of strains) (Supplementary Table S4). Of the total gene clusters, 202 (2.9%) were detected exclusively in DLB3 within the dataset (Figure 9), most of which were annotated as encoding hypothetical proteins (Supplementary Table S5). Notably, loci APBKEJPL_03128–03151 corresponded to an 83.8-kb hybrid NRPS/*trans*-AT PKS/lanthipeptide region (antiSMASH region 5.2) with no match to characterized clusters in the MIBiG database, representing an uncatalogued DLB3-specific biosynthetic locus of unknown product.

Comparative antiSMASH analysis of the same ten genomes revealed that biosynthetic gene clusters associated with fengycin, bacillaene, bacilysin, bacillibactin, and surfactin were conserved in DLB3 and most reference strains, including the well-characterized biocontrol strain *B. velezensis* FZB42 (Supplementary Table S6). Conversely, the plantazolicin-associated cluster detected in FZB42 was not identified in DLB3. The andalusicin A/B-associated cluster was detected in DLB3 but not in the nine reference genomes, representing a distinguishing feature of DLB3 within the comparative dataset. Overall, comparative genomic analyses showed that DLB3 shares several major secondary metabolite biosynthetic gene clusters with other *B. velezensis* strains, including FZB42, while also possessing distinct genomic features.

## 3. Discussion

Determining the bacterium’s exact taxonomic position provides a critical framework for interpreting its biological potential. However, species within the *Bacillus subtilis* complex are difficult to distinguish using conventional approaches, including morphology, biochemical tests, and 16S rRNA gene analysis, because these species share similar traits and have highly conserved 16S rRNA sequences [9,33]. Recently, the development of high-throughput whole-genome sequencing has greatly improved taxonomic classification by providing robust genomic markers for precise species delineation [34]. In this study, the draft genome of strain DLB3, which was isolated from the black pepper rhizosphere, was sequenced and assembled. Phylogenomic and comparative genomic analyses clearly classified DLB3 as *Bacillus velezensis*. Strain DLB3 showed ANI values above 97% and dDDH values above 79% compared with genomes of eight *B. velezensis* strains, including the type strain NRRL B-41580, exceeding the widely accepted species delineation thresholds of 95–96% for ANI and 70% for dDDH [20,21,22].

The antagonistic activity of strain DLB3 against fungal and bacterial phytopathogens is consistent with the diverse array of biosynthetic gene clusters identified in its genome. *B. velezensis* is widely recognized for producing multiple classes of antimicrobial lipopeptides and polyketides including surfactin, fengycin, bacillaene, macrolactin, and difficidin [35]. The presence of these clusters in the DLB3 genome may therefore contribute to its observed antagonistic potential. In some strains, such clusters account for over 10% of the genome, as observed in FZB42 and K1 [2], reflecting a substantial genomic investment in secondary metabolite production. The conservation of core clusters (fengycin, bacilysin, bacillibactin) alongside accessory clusters unique to DLB3 or shared with only one other strain (Section 2.9) reflects the open pan-genomic structure characteristic of *B. velezensis*, in which a conserved core genome coexists with a variable accessory genome shaping niche-specific versatility [5,36]. Comparative genomic analysis further distinguished DLB3 from closely related *B. velezensis* strains, including the well-characterized biocontrol strain FZB42, by identifying an andalusicin A/B biosynthetic gene cluster exclusive to DLB3 and a putative hybrid NRPS/PKS/lanthipeptide region absent from all reference genomes examined. These findings suggest that DLB3 harbors distinct accessory biosynthetic features in addition to the core antimicrobial clusters shared with other *B. velezensis* strains, although the metabolites they produce and their functional significance remain to be determined. In addition, the presence of genes encoding putative cell wall-degrading enzymes, including chitinases, glucanases, and lysozyme-like proteins, in the DLB3 genome suggests that these enzymes may contribute to its antagonistic activity, potentially complementing antibiotic-mediated mechanisms [37,38].

In the present study, DLB3 produced an inhibition zone of 8.4 mm against *R. solanacearum*, lower than the 20.40 mm reported for FZB42 against the same pathogen [39]. Nevertheless, this value falls within the range reported for other *B. velezensis* isolates (7.00–20.40 mm), with comparable values observed for strains F7 (8 mm) [40] and OR1-39 (7.00 mm) [39]. Against fungal pathogens, DLB3 exhibited PIRG values of 47.6–71.2% against *F. falciforme*, *F. oxysporum*, and *C. gloeosporioides*. These values exceeded those reported for *B. velezensis* FB2 against *F. oxysporum* (40.4%) and *F. solani* (26.0%) [41], although differences in target species and experimental conditions limit direct comparisons. Overall, DLB3 exhibited pathogen-dependent antagonistic activity, with the culture supernatant showing antibacterial activity against *R. solanacearum* and the dual-culture assay revealing variable levels of fungal growth inhibition. However, the dual-culture assay did not distinguish between contact-dependent inhibition and inhibition mediated by diffusible compounds; therefore, the mechanisms underlying the observed antifungal activity remain to be elucidated.

The broad tolerance of strain DLB3 to temperature (25–40 °C optimal, with residual growth up to 50 °C), pH (5–9), and salinity (up to 10% NaCl) is comparable to other rhizosphere-derived *B. velezensis* strains, such as CM5 (pH 5.0–8.5, up to 9% NaCl, 15–40 °C) [17] and C3-3 (growth at 50 °C, up to 10% NaCl) [18], suggesting adaptive traits that may contribute to rhizosphere competence in PGPR. Genes associated with Cu, Zn, and Cr resistance, with sequence identities of up to 96.5%, 51.8%, and 61.7%, respectively, provide a plausible genetic basis for the metal tolerance observed in DLB3. For lead, only one putative resistance-associated gene, *nmtR*, was identified, with a moderate sequence identity of 45.5%. Nevertheless, DLB3 showed its highest MIC for Pb (1800 mg L^−1^) among the tested metals. This difference between the phenotypic tolerance and the identified genetic determinants indicates that the mechanisms underlying Pb tolerance in DLB3 may be more complex than suggested by the identified determinants. This pattern is consistent with previous reports showing that bacterial Pb tolerance is frequently mediated, at least in part, by passive, gene-independent mechanisms such as extracellular biosorption and precipitation, whereby Pb^2+^ ions are immobilized by negatively charged functional groups (specifically carboxyl, hydroxyl, and phosphate groups) on the cell wall and extracellular polymeric substances [42,43]. However, the metal tolerance observed under laboratory conditions should not be directly extrapolated to agricultural soils, where metal bioavailability is influenced by soil properties and chemical speciation [44].

The susceptibility of strain DLB3 to all nine clinically relevant antibiotics tested supports its favorable biosafety profile for potential agricultural application. Genome-wide screening against the CARD database further corroborated this profile: most resistance determinants corresponded to antibiotics outside the tested panel or to disinfecting agents, while incomplete, low-identity vancomycin resistance gene homologs matched the observed susceptible phenotype. One notable exception was the detection of a high-identity (97.4%) homolog of the *cfr*-like 23S rRNA methyltransferase gene, annotated as *clbA*. A similar observation was reported for *B. velezensis* DSM SNR14-4, in which a *clbA* was detected in the genome, while phenotypic susceptibility testing did not indicate resistance to the antibiotics associated with this determinant [45]. Similarly, a *clbA* homolog with 97% sequence identity was detected in *B. velezensis* DSM 15544 but was not considered a safety concern because it was found in the chromosome, as opposed to a bacterial plasmid [46]. Nonetheless, given the known potential for horizontal transfer of *cfr*-family genes among Gram-positive bacteria [47], transcriptional and functional characterization of this locus is warranted before large-scale field application of strain DLB3.

Several findings in this study remain predictive and were evaluated primarily under in vitro conditions. In particular, genome-mining results should not be interpreted as direct evidence of functional activity, as sequence similarity alone does not establish gene expression or phenotypic effects. This is illustrated by the genotype–phenotype discordance observed for the *cfr*-like determinant, which was detected in the genome despite the absence of the corresponding phenotypic resistance under the conditions tested. Similarly, comparative genomic analysis identified candidate genetic features that distinguish DLB3 from closely related strains, but their contribution to biocontrol activity has not been experimentally established. Therefore, targeted functional and gene-expression studies are needed to clarify the contribution of key genomic determinants to the observed traits. In addition, in planta and greenhouse or field experiments will be necessary to determine whether the antagonistic and plant-associated traits observed in vitro are maintained under more complex agricultural conditions and to further evaluate the efficacy and biosafety of DLB3.

## 4. Materials and Methods

### 4.1. Strains

*Bacillus* sp. DLB3 was isolated from the rhizosphere soil of black pepper (*Piper nigrum* L.) plants growing in Dak Lak province, Vietnam. *Fusarium falciforme* strain and *Fusarium oxysporum* strain were recovered from the roots of diseased black pepper plants collected in Dak Lak province, Vietnam. *Colletotrichum gloeosporioides* strain was obtained from diseased avocado leaves collected in Lam Dong province, Vietnam. *Ralstonia solanacearum* strain was isolated from the roots of diseased tomato plants collected in Dong Nai province, Vietnam. All bacterial and fungal strains were maintained in 20% (v/v) glycerol at −80 °C until further use.

### 4.2. Morphological Analysis

The colony characteristics of strain DLB3 were recorded after incubation on LB agar plates at 37 °C for 24 h. Gram staining was performed on an overnight culture of strain DLB3 following standard procedures. The Schaeffer–Fulton method was used to observe endospore formation [48]. Cell morphology was examined by scanning electron microscopy (JSM-IT200, JEOL, Tokyo, Japan). Briefly, cells from 24-h cultures were harvested by centrifugation (HERMLE Z 323 K, Gosheim, Germany) at 4000 rpm for 10 min at 4 °C and fixed in 2.5% glutaraldehyde overnight, followed by washing with 0.1 M phosphate buffer. The samples were dehydrated through a graded ethanol series (30, 50, 70, 80, 90, and 100%), treated with tert-butyl alcohol for 30 min, air-dried on a glass coverslip, coated with platinum, and observed under SEM.

### 4.3. Genome Sequencing, Assembly, and Annotation

Genomic DNA of strain DLB3 was extracted from bacterial colonies grown on LB agar using the QIAamp DNA Mini kit (Qiagen, Germantown, MD, USA) according to the manufacturer’s instructions. Whole-genome sequencing libraries were prepared using the NEBNext dsDNA Fragmentase and the NEBNext Ultra II DNA Library Prep Kit for Illumina (New England Biolabs, Ipswich, MA, USA). Whole-genome sequencing was performed using the Onso sequencing platform (PacBio, Menlo Park, CA, USA) with sequencing-by-binding chemistry to generate 150-bp paired-end reads. Quality control and preprocessing of the raw sequencing reads were performed using fastp v0.23.1 [49] for adapter removal, trimming of low-quality bases, and filtering of reads containing ambiguous nucleotides. Quality-filtered reads were assembled de novo with Unicycler v0.4.8 [50]. The resulting assemblies were evaluated using QUAST v5.2.0 [51] and reads were subsequently realigned to the assembled contigs to identify regions with insufficient read support, potentially reflecting assembly errors. Genome completeness and contamination levels were assessed with CheckM v1.2.1 [52]. The genome sequence of strain DLB3 has been deposited in GenBank under accession number JCDZAX000000000, within BioProject PRJNA1530694. Genome annotation was conducted with Prokka v1.14.6 [53]. The distribution of predicted genes into Clusters of Orthologous Groups (COGs) was determined using eggNOG-mapper v2.1.13 [54] on the Galaxy platform (https://usegalaxy.eu/ (accessed on 27 February 2026)) [55], and functional categories were assigned based on the COG classification system. The relative abundance of each COG category was calculated based on the total number of annotated genes.

### 4.4. Phylogenomic Analysis and Taxonomic Classification

The genome of strain DLB3 was compared with those of 14 reference genomes retrieved from the NCBI database, including eight representative strains of *Bacillus velezensis* (comprising the type strain NRRL B-41580) and the type strains of six other closely related *Bacillus* species (*Bacillus amyloliquefaciens*, *Bacillus siamensis*, *Bacillus nakamurai*, *Bacillus subtilis*, *Bacillus cabrialesii*, and *Bacillus tequilensis*). Pan-genome analysis was conducted using Roary v3.13.0 [56] with a BLASTp minimum percentage identity threshold of 95%, and a concatenated core-gene multiple sequence alignment was used for phylogenetic reconstruction. A maximum-likelihood tree was inferred using IQ-TREE 2 [57], with the best-fit substitution model selected using ModelFinder, and branch support was assessed with 1000 ultrafast bootstrap replicates. The resulting tree was visualized and annotated using iTOL v6 (https://itol.embl.de/ (accessed on 21 August 2026)) [58].

Digital DNA–DNA hybridization (dDDH) values were calculated using the Genome BLAST Distance Phylogeny (GBDP) algorithm implemented in the Genome-to-Genome Distance Calculator (GGDC) v3.0 web platform (https://ggdc.dsmz.de/ (accessed on 24 August 2026)) [59], based on formula 2. Average nucleotide identity (ANI) was calculated using the JSpeciesWS web server (https://jspecies.ribohost.com/jspeciesws/ (accessed on 24 August 2026)) [60]. Heatmaps of ANI and dDDH values were generated using the ggplot2 package [61] in the R environment [62].

### 4.5. Antagonistic Activity of Bacillus sp. DLB3

The antagonistic activity of strain DLB3 against *F. falciforme*, *F. oxysporum*, and *C. gloeosporioides* was evaluated using a dual-culture assay [63]. A mycelial plug (≈5 mm in diameter) was taken from a culture grown for 48 h and placed in the centre of a potato dextrose agar (PDA) plate. Strain DLB3 was streaked in two straight lines 2.5 cm from the plug. Plates inoculated with the mycelial plug and sterile water were used as control. The plates were incubated at 28 °C for 7 days, with three replicate plates for each pathogen. The inhibitory effect of strain DLB3 was determined as the percentage inhibition of radial growth (PIRG) based on fungal colony diameter, using the following equation:

(1) $$\mathrm{PIRG}\;(\%)\;=[1-\left(\frac{D1}{D0}\right)]\times 100$$

where D1 is the colony diameter in the presence of strain DLB3 and D0 is the colony diameter in the control.

The antibacterial activity of strain DLB3 against *R. solanacearum* was determined using a disc diffusion assay [64]. Strain DLB3 was cultured in LB broth at 37 °C for 48 h with shaking at 120 rpm, then centrifuged at 8000 rpm for 10 min at 4 °C, and the supernatant was filter-sterilized through a 0.22 μm membrane to obtain the cell-free supernatant. An overnight culture of *R. solanacearum* grown in LB broth was adjusted to 0.5 McFarland standard was evenly streaked onto the surface of Mueller–Hinton agar (MHA) plates using a sterile cotton swab. Sterile filter paper discs (6 mm in diameter) loaded with 20 μL of the DLB3 cell-free supernatant were placed on the inoculated plates. A tetracycline disc (30 μg) served as the positive control, while sterile LB broth served as the negative control. The assay was performed in triplicate. Plates were incubated at 28 °C for 24 h and antibacterial activity was determined by measuring the diameter of the inhibition zone (mm).

### 4.6. Production of Lytic Enzymes

Extracellular amylase, protease, and CMCase activities of strain DLB3 were assessed by spot inoculation of 2 μL of an overnight culture adjusted to 0.5 McFarland onto substrate-specific agar media.

Strain DLB3 was inoculated onto starch agar medium containing (g L^−1^), soluble starch 10 g, tryptone 10 g, glucose 5 g, NaCl 5 g, beef extract 5 g, and agar 18 g (pH 7.0). After incubation at 37 °C for 48 h, the plates were flooded with Lugol’s iodine solution (1% iodine in 2% potassium iodide (w/v)), and clear zones surrounding the bacterial growth were recorded as evidence of starch hydrolysis [65].

The ability to produce CMCase was determined by spot inoculation on CMC agar plates containing (g L^−1^), CMC 2 g, NaNO_3_ 1 g, K_2_HPO_4_ 1 g, KCl 1 g, MgSO_4_ 0.5 g, yeast extract 5 g, and agar 15 g (pH 7.0) [66]. After incubation at 37 °C for 48 h, the plates were flooded with Lugol’s iodine solution [67]. CMC hydrolysis was visualized as a clear zone surrounding the bacterial growth.

Proteolytic activity was evaluated on casein agar medium containing (g L^−1^), casein 10 g, K_2_HPO_4_ 2 g, glucose 1 g, peptone 5 g, and agar 15 g (pH 7.0). The plates were incubated at 37 °C for 48 h. Following incubation, the plates were flooded with 25% (w/v) trichloroacetic acid and maintained at 45 °C for 15 min to precipitate unhydrolyzed proteins. The formation of a clear zone surrounding the colony was taken as evidence of extracellular protease activity [68]. All assays were performed in triplicate, and the diameter of the clear hydrolysis zone, including the colony, was measured. Substrate-specific agar plates without strain DLB3 inoculation served as the control.

### 4.7. Determination of Catecholate-Type Siderophore Production

The ability to produce catecholate-type siderophores was assessed using the Arnow method. Strain DLB3 was grown on LB broth at 37 °C for 24 h. The cells were then harvested by centrifugation at 4000 rpm for 10 min at 4 °C and washed twice with minimal medium (MM) broth (10 g/L glucose, 1.47 g/L glutamic acid, 3.0 g/L K_2_HPO_4_, 1.0 g/L KH_2_PO_4_, 0.5 g/L NH_4_Cl, 0.1 g/L NH_4_NO_3_, 0.1 g/L Na_2_SO_4_, 10 mg/L MgSO_4_·7H_2_O, 1 mg/L MnSO_4_·4H_2_O, and 0.5 mg/L CaCl_2_). The washed cells were resuspended in the same medium and was cultured in 100 mL of MM broth (initial OD_600_ ≈ 0.1) at 37 °C for 48 h. A parallel culture was performed using MM broth supplemented with 29 mg/L iron(III) chloride (FeCl_3_) [69]. Uninoculated MM broth, processed under the same conditions, served as the control.

The Arnow assay was used to detect catechol-type siderophores based on the colorimetric response of catechol compounds to a nitrite molybdate reagent. Under acidic conditions, the reaction mixture develops a yellow color, which turns orange-red following alkalinization. Briefly, 1.0 mL of culture filtrate was mixed with 1.0 mL of 0.5 mol L^−1^ HCl, followed by the addition of 1.0 mL of the nitrite molybdate reagent and 1.0 mL of 1.0 mol L^−1^ NaOH. The mixture was kept at room temperature for approximately 5 min to allow color development. Deionized water (1.0 mL) was used as the blank. The nitrite molybdate reagent was prepared by dissolving 10 g each of sodium nitrite and sodium molybdate in 100 mL of deionized water. The formation of an orange-red color was taken as a positive indication of catecholate-type siderophore production, with color intensity increasing according to the amount of catechol present [69].

### 4.8. Evaluation of Tolerance to Abiotic Stresses and Heavy Metals

The growth of strain DLB3 under different temperatures (15–55 °C), pH values (4–10), and salt concentrations (1–12%) was measured to evaluate its tolerance to these abiotic stresses [70]. An overnight culture of strain DLB3 (adjusted to a 0.5 McFarland standard) was inoculated at 0.5 mL into 50 mL of LB broth. For the temperature assay, cultures were incubated at 15, 20, 25, 30, 35, 40, 45, 50, or 55 °C at pH 7. For the pH assay, cultures were incubated at 37 °C in LB broth adjusted to pH 4, 5, 6, 7, 8, 9, or 10. For the salinity assay, cultures were incubated at 37 °C and pH 7 in LB broth containing final NaCl concentrations of 1, 2, 4, 6, 8, 10, or 12%. All cultures were incubated at 120 rpm for 24 h in triplicate, and growth was determined by measuring the optical density at 600 nm (OD_600_).

The tolerance of strain DLB3 to four heavy metals was assessed by determining the minimum inhibitory concentrations (MICs) using an agar dilution assay with minor modifications [71]. Lead nitrate [Pb(NO_3_)_2_], zinc sulfate (ZnSO_4_), copper sulfate (CuSO_4_), and potassium dichromate (K_2_Cr_2_O_7_) were used as sources of Pb(II), Zn(II), Cu(II), and Cr(VI), respectively. An overnight culture of strain DLB3, adjusted to a 0.5 McFarland standard, was spot-inoculated (2 μL) onto MHA medium supplemented with increasing concentrations of Pb (1600, 1650, 1700, 1750, and 1800 mg L^−1^), Cr (100, 150, 200, 250, and 300 mg L^−1^), Cu (250, 300, 350, 400, and 450 mg L^−1^), and Zn (300, 350, 400, 450, and 500 mg L^−1^). These concentration ranges were selected based on preliminary screening across a broad range of metal concentrations (100–3200 mg L^−1^), followed by stepwise refinement of the concentration ranges to determine the MIC of each metal. Stock solutions of the metal salts were sterilized by filtration through 0.22 μm membranes before being added to the medium. MHA plates without heavy metal supplementation served as the control. All treatments were performed in triplicate. Plates were incubated at 37 °C for 48 h, and bacterial growth was assessed visually. The MIC was defined as the lowest metal concentration that completely inhibited visible growth.

### 4.9. Antimicrobial Susceptibility Testing

Antimicrobial susceptibility testing was performed using the EUCAST standardized disk diffusion method. Briefly, bacterial colonies from an overnight culture were suspended in sterile saline and adjusted to 0.5 McFarland standard. The suspension was evenly inoculated over the entire surface of Mueller–Hinton agar (MHA) plates using a sterile cotton swab. Antibiotic disks, along with a blank sterile disk as the negative control, were placed onto the inoculated agar surface within 15 min of inoculation. The plates were incubated in air at 35 ± 1 °C for 18 ± 2 h, in triplicate. After incubation, the diameters of the inhibition zones were measured in millimetres, and results were interpreted according to the EUCAST clinical breakpoint criteria for *Bacillus* spp. (excluding *Bacillus anthracis*) [72].

### 4.10. Genome Mining for Functional Gene Clusters and Resistance Determinants

Secondary metabolite biosynthetic gene clusters were identified using antiSMASH v8.0.4 (https://antismash.secondarymetabolites.org/ (accessed on 21 August 2026)) [73]. Carbohydrate-active enzymes (CAZymes) were annotated using the dbCAN3 (https://pro.unl.edu/dbCAN2/ (accessed on 4 September 2026)) [74], which integrates multiple tools (HMMER, DIAMOND, and dbCAN-sub) for CAZymes prediction; only genes supported by at least two tools were retained as reliable annotations. Genes encoding peptidases were identified by BLASTp (v2.17.0) [75] of the predicted proteome against the MEROPS peptidase database [76], using a minimum identity of 30%, query coverage of 50%, and an E-value threshold of 1 × 10^−5^; for each locus, the best-matching MEROPS entry was retained based on the highest bit score. The genome was similarly screened against the BacMet experimentally confirmed database (v2.0) [77] using BLASTp [75], applying a minimum amino-acid identity of 30%, query coverage of 70%, and an E-value threshold of 1 × 10^−5^, with hits classified according to their associated metal based on BacMet gene annotations. Genome-wide screening for antibiotic resistance genes was performed using the Resistance Gene Identifier (RGI) via the CARD web platform (https://card.mcmaster.ca/analyze/rgi (accessed on 7 September 2026)) [78], applying the “Strict” hit criteria against the CARD database.

### 4.11. Statistical Analysis

Data are presented as mean ± standard deviation (SD) from three replicates. Differences among groups were analyzed using one-way analysis of variance (ANOVA), followed by Tukey’s honestly significant difference (HSD) test for multiple comparisons. Statistical significance was set at *p* < 0.05.

## 5. Conclusions

This study characterized strain DLB3, a rhizobacterium isolated from black pepper rhizosphere soil, and confirmed its identity as *Bacillus velezensis* through phylogenomic placement and species-delineation genomic indices (ANI, dDDH). The strain displayed antagonistic activity against fungal and bacterial phytopathogens, together with siderophore and extracellular enzyme production, broad physiological tolerance to temperature, pH, and salinity, resistance to multiple heavy metals, and a favorable antibiotic susceptibility profile. Genome mining identified biosynthetic gene clusters, carbohydrate-active enzymes, peptidases, and metal resistance determinants that provide a plausible genetic basis for several of these phenotypic traits. Collectively, these findings support the potential of strain DLB3 as a multifunctional biocontrol candidate with tolerance to abiotic stresses and antibiotic-susceptible biosafety traits, warranting further functional and field-based validation prior to practical application.

## Figures and Tables

**Table 1:** De novo genome assembly statistics of strain DLB3.

Attribute	Value
Genome size (bp)	3,913,361
Number of contigs	44
G+C content (bp)	46.45%
Genes (total)	3818
CDSs (total)	3740
The longest contig (bp)	1,115,001
tRNAs	74
rRNAs	3
N50 value	990,239
L50 value	2

N50 is defined as the shortest contig length such that the sum of contigs of equal or greater length covers 50% of the total assembly length. L50 is defined as the smallest number of contigs whose combined length accounts for at least 50% of the genome size.

**Table 2:** Antimicrobial susceptibility profile of strain DLB3 according to EUCAST breakpoints.

Antibiotic Class	Antibiotic (Disk Content)	Zone Diameter (mm, Mean ± SD)	Interpretation
Carbapenems	Meropenem (10 µg)	29.7 ± 0.7	S
	Imipenem (10 µg)	38.2 ± 1.4	S
Fluoroquinolones	Ciprofloxacin (5 µg)	23.7 ± 1.4	I
	Levofloxacin (5 µg)	27.6 ± 1.3	I
	Norfloxacin (10 µg, screen only)	25.3 ± 0.5	Screen-negative
Glycopeptides	Vancomycin (5 µg)	17.5 ± 0.3	S
Macrolides and lincosamides	Erythromycin (15 µg)	26.2 ± 1.3	S
	Clindamycin (2 µg)	26.7 ± 1.5	S
Oxazolidinones	Linezolid (10 µg)	25.6 ± 1.0	S

S: susceptible; I: susceptible, increased exposure (based on negative norfloxacin screen per EUCAST Note B).

**Table 3:** Biosynthetic gene clusters predicted in the genome of strain DLB3 and their distribution in related *Bacillus* genomes.

*B. velezensis* DLB3				
Region	Cluster Type	Most Similar Known Cluster (MIBiG)	Similarity Confidence	Predicted Function
1.1	NRPS, betalactone, transAT-PKS	Fengycin	High	Antifungal [23]/Antibacterial activity [24]
1.2	transAT-PKS, NRPS, T3PKS	Bacillaene	High	Antibacterial activity [25]
1.3	trans-AT PKS	Macrolactin H	High	Antibacterial [26]
1.4	terpene	Unknown	Unknown	Unknown
1.5	PKS-like	Unknown	Unknown	Unknown
2.1	Other	Bacilysin	High	Antibacterial [12]/Antifungal activity [27]
2.2	RiPP-like, NRP-metallophore, NRPS, terpene-precursor	Bacillibactin	High	Siderophore [28]
3.1	NRPS	Bacillothiazol A-N	High	Protein tyrosine phosphatase PTP1B inhibitory activity [29]
3.2	terpene-precursor	Unknown	Unknown	Unknown
4.1	terpene	Unknown	Unknown	Unknown
4.2	T3PKS	Unknown	Unknown	Unknown
4.3	transAT-PKS-like	Difficidin	Medium	Antibacterial activity [12]
5.1	NRPS	Surfactin	High	Antiviral [30]/Antibacterial activity [31]
5.2	NRPS/RiPP hybrid	Andalusicin A/B	High	Antibacterial activity [32]
10.1	transAT-PKS	Difficidin	Low	Antibacterial activity [12]
11.1	transAT-PKS-like	Difficidin	Low	Antibacterial activity [12]
13.1	NRPS	Fengycin	Low	Antifungal activity [23]
14.1	NRPS	Unknown	Unknown	Unknown

## Data Availability

The genome sequence of strain DLB3 has been deposited in GenBank under accession number JCDZAX000000000, within BioProject PRJNA1530694.

## Supplementary Materials

The following supporting information can be downloaded at: https://www.mdpi.com/article/10.3390/ijms27219536/s1.

## Abbreviations

The following abbreviations are used in this manuscript:

ANI	Average nucleotide identity
BGCs	Biosynthetic gene clusters
BLASTp	Basic local alignment search tool for proteins
CDSs	Coding sequences
CMC	Carboxymethyl cellulose
CMCase	Carboxymethyl cellulase
COG	Clusters of orthologous groups
dDDH	Digital DNA–DNA hybridization
EUCAST	European Committee on Antimicrobial Susceptibility Testing
HSD	Honestly significant difference
LB	Luria–Bertani
MHA	Mueller–Hinton agar
MIBiG	Minimum information about a biosynthetic gene cluster
MICs	Minimum inhibitory concentrations
MM	Minimal medium
NCBI	National Center for Biotechnology Information
PGPR	Plant growth-promoting rhizobacteria
PIRG	Percentage inhibition of radial growth
SD	Standard deviation
