# Supplementary material for: Phenotypic and Genomic Characterization of Bacillus sp. DLB3, a Black Pepper Rhizosphere Isolate with Antagonistic, Abiotic Stress-Tolerant, and Antibiotic-Susceptible Traits
Source: Int J Mol Sci. 2026 Oct 25;27(21):9536. doi: 10.3390/ijms27219536 (PMC13762528; doi:10.3390/ijms27219536)
Supplement: Supplementary file 1 [file ijms-27-09536-s001.zip › ijms-4604862-supplementary.pdf]

Table S1. Peptidase-encoding genes identified in the genome of strain DLB3

DLB3 locus tag	MEROPS ID	Annotation	Family	Identity (%)	Query coverage (%)	E-value	Bit score
APBKEJPL_00866	MER0000311	subtilisin BPN' (*Bacillus amyloliquefaciens*)	S08	97.8	72	0	535
APBKEJPL_03622	MER0001242	subfamily M24A unassigned peptidases (*Bacillus subtilis*)	NA	96.8	100	0	500
APBKEJPL_00378	MER0001038	bacillolysin (*Bacillus* sp.)	M04	95.7	58.2	0	605
APBKEJPL_03087	MER0001424	pyroglutamyl-peptidase I (prokaryote) (*Bacillus amyloliquefaciens*)	C15	95.3	100	5.28e-155	424
APBKEJPL_00525	MER0000320	IspA peptidase (*Bacillus subtilis*)	S08	88.8	90	0	526
APBKEJPL_02432	MER0001291	gpr peptidase (*Bacillus subtilis*)	A25	85.5	95.4	0	627
APBKEJPL_03203	MER0001513	glutamyl peptidase BS (*Bacillus subtilis*)	S01	83.9	69.6	5.32e-125	352
APBKEJPL_01123	MER0001567	HtrA-like peptidase ({*Listeria*}-type) (*Bacillus subtilis*)	S01	80.8	71.9	5.46e-176	488
APBKEJPL_03782	MER0000447	D-Ala-D-Ala carboxypeptidase A (*Bacillus subtilis*)	S11	80.8	93	0	649
APBKEJPL_01660	MER0001442	peptidase Clp (type 1) (*Clostridium beijerinckii*)	S14	71.3	94.9	1.50e-101	287
APBKEJPL_00417	MER0001511	signal peptidase SipT (*Bacillus amyloliquefaciens*)	S26	99.4	80.8	1.01e-114	319
APBKEJPL_02894	MER0000593	signal peptidase SipS (*Bacillus amyloliquefaciens*)	S26	99.3	79	8.82e-109	303
APBKEJPL_00077	MER0000586	repressor LexA (*Bacillus subtilis*)	S24	92	54.4	1.95e-73	213
APBKEJPL_00239	MER0001626	CodW component of CodWX peptidase (*Bacillus subtilis*)	T01	92	96.7	5.33e-119	330
APBKEJPL_02226	MER0000391	Other protein (*Bacillus subtilis*)	M42	90.5	98.9	0	674
APBKEJPL_02909	MER0000454	D-Ala-D-Ala carboxypeptidase DacF (*Bacillus subtilis*)	S11	86.3	91.8	0	623
APBKEJPL_00303	MER0001496	family M38 unassigned peptidases (*Bacillus subtilis*)	NA	83.2	73.8	0	539

APBKEJPL_01979	MER0000458	penicillin-binding protein 2 (*Bacillus subtilis*)	X52	83.2	60.1	0	661
APBKEJPL_02882	MER0000456	K15-type DD-transpeptidase (*Bacillus subtilis*)	S11	79.7	92.4	0	594
APBKEJPL_00322	MER0001293	sporulation factor SpoIIGA (*Bacillus subtilis*)	A36	71.6	52.4	7.70e-85	248
APBKEJPL_02585	MER0000473	D-Ala-D-Ala carboxypeptidase ({Actinomadura}-type) (*Bacillus subtilis*)	S13	62.2	92.1	0	596
APBKEJPL_00175	MER0001659	family S14 unassigned peptidases (*Clostridium beijerinckii*)	NA	60.8	61.4	2.17e-63	191
APBKEJPL_02303	MER0000869	ComC peptidase (*Bacillus subtilis*)	A24	58.3	60.4	3.34e-59	180
APBKEJPL_02113	MER0001356	subfamily M20A unassigned peptidases (*Geobacillus stearothermophilus*)	NA	55.5	97	0	514
APBKEJPL_01245	MER0001421	peptidase T (*Escherichia coli*)	M20	54.8	96.6	3.12e-155	440
APBKEJPL_00846	MER0000596	subfamily S26A unassigned peptidases (*Bacillus caldolyticus*)	NA	54.3	81.9	1.62e-48	150
APBKEJPL_01720	MER0001289	YwaD peptidase (*Bacillus subtilis*)	M28	54.3	62.7	7.21e-94	280
APBKEJPL_00308	MER0001498	family A8 unassigned peptidases (*Staphylococcus carnosus*)	NA	52.7	98	1.28e-49	152
APBKEJPL_02559	MER0000108	SpoIVB peptidase (*Clostridium cellulolyticum*)	S55	52.5	51.9	1.41e-73	226
APBKEJPL_03368	MER0001835	family C26 unassigned peptidases (*Sanguibacter keddieii*)	NA	52.4	74	1.18e-61	187
APBKEJPL_00413	MER0001287	aminopeptidase II ({*Bacillus*}-type) (*Geobacillus stearothermophilus*)	M29	52.2	100	1.04e-165	467
APBKEJPL_02124	MER0000391	Other protein (*Bacillus subtilis*)	M42	50.3	99.2	6.87e-130	372
APBKEJPL_00406	MER0001490	family M38 non-peptidase homologues (*Clostridium beijerinckii*)	NA	50.2	57.4	1.39e-120	355
APBKEJPL_01633	MER0001444	imidazole glycerol phosphate synthase subunit HisH (*Clostridium beijerinckii*)	C26	50	97.2	1.71e-66	200
APBKEJPL_03366	MER0001670	amidophosphoribosyltransferase precursor (*Clostridium beijerinckii*)	C44	49.2	50	1.13e-81	249

APBKEJPL_03780	MER0001573	imidazoleglycerol-phosphate synthase ({*Pyrococcus furiosus*}) (*Halomicrobium mukohataei*)	NA	48.7	95.4	7.74e-52	161
APBKEJPL_00183	MER0001660	BSSC8_26020 g.p. ({*Bacillus subtilis*}) (*Clostridium beijerinckii*)	NA	47.9	52.8	1.73e-71	220
APBKEJPL_03456	MER0001249	SPBPB2B2.05 ({*Schizosaccharomyces pombe*}) (*Fusobacterium nucleatum*)	NA	47.2	96.2	1.35e-77	230
APBKEJPL_00555	MER0001567	HtrA-like peptidase ({*Listeria*}-type) (*Bacillus subtilis*)	S01	46.6	64.4	3.00e-80	246
APBKEJPL_00887	MER0002011	family M20D unassigned peptidases (*Geobacillus stearothermophilus*)	NA	46.4	77	4.83e-96	287
APBKEJPL_01832	MER0001567	HtrA-like peptidase ({*Listeria*}-type) (*Bacillus subtilis*)	S01	45.9	62.5	5.83e-82	250
APBKEJPL_02499	MER0001505	gamma-D-glutamyl-(L)-meso-diaminopimelate peptidase I (*Lysinibacillus sphaericus*)	M14	44.6	78.9	3.41e-89	266
APBKEJPL_02679	MER0000280	subfamily M15B unassigned peptidases (*Bacillus cereus*)	NA	44.2	57.4	4.82e-34	117
APBKEJPL_03260	MER0001676	PfpI peptidase (*Paraburkholderia fungorum*)	C56	43.9	66.7	6.00e-26	92.4
APBKEJPL_02536	MER0001458	subfamily M24B unassigned peptidases (*Chloroflexus aurantiacus*)	NA	43.4	64	3.51e-62	194
APBKEJPL_02374	MER0001668	collagenase ({*Helicobacter*}-type) (*Clostridium beijerinckii*)	U32	42.7	91	3.64e-114	335
APBKEJPL_02167	MER0001302	subfamily S49A unassigned non-peptidase homologues (*Leptospira borgpetersenii*)	NA	42	61.2	5.84e-46	152
APBKEJPL_03492	MER0000501	family M20 non-peptidase homologues (*Agrobacterium tumefaciens*)	NA	40.3	90.4	1.71e-94	285
APBKEJPL_02776	MER0001186	carboxypeptidase Taq (*Thermus aquaticus*)	M32	40.2	97.6	9.23e-125	370
APBKEJPL_02012	MER0001676	PfpI peptidase (*Paraburkholderia fungorum*)	C56	40	80.5	2.23e-33	111

APBKEJPL_01074	MER0000319	minor extracellular protease ({*Bacillus licheniformis*}) (*Bacillus subtilis*)	S08	39.8	83.3	2.34e-63	198
APBKEJPL_01623	MER0001875	family M38 non-peptidase homologues (*Ketogulonicigenium vulgare*)	NA	39.7	80.1	3.65e-50	167
APBKEJPL_02592	MER0001978	gamma-glutamyltransferase 1 (bacterial-type) (*Escherichia coli*)	T03	39.6	92.7	6.95e-130	387
APBKEJPL_01378	MER0001685	subfamily M50B unassigned peptidases (*Clostridium beijerinckii*)	NA	39.1	77.2	1.58e-36	123
APBKEJPL_00469	MER0001458	subfamily M24B unassigned peptidases (*Chloroflexus aurantiacus*)	NA	38.9	62.1	2.58e-55	177
APBKEJPL_00129	MER0000317	subtilisin lentus (*Bacillus lentus*)	S08	38.6	66.7	3.05e-45	154
APBKEJPL_01510	MER0000567	oxamate amidohydrolase proenzyme ({*Klebsiella pneumonia*}) (*Halomicrobium mukohataei*)	T03	38.1	95	3.25e-87	274
APBKEJPL_03494	MER0002007	carboxypeptidase Ss1 (*Sulfolobus solfataricus*)	M20	38	96.9	2.23e-78	243
APBKEJPL_02995	MER0002007	carboxypeptidase Ss1 (*Sulfolobus solfataricus*)	M20	37.8	93.2	3.44e-87	265
APBKEJPL_00436	MER0000982	family M20D unassigned peptidases (*Clostridium beijerinckii*)	NA	36.9	101.3	8.66e-74	230
APBKEJPL_03275	MER0001242	subfamily M24A unassigned peptidases (*Bacillus subtilis*)	NA	36.5	100	5.01e-53	168
APBKEJPL_00504	MER0000983	subfamily M48B unassigned peptidases (*Halomicrobium mukohataei*)	NA	35.6	70	2.86e-23	90.5
APBKEJPL_02149	MER0001676	PfpI peptidase (*Paraburkholderia fungorum*)	C56	35.5	81.7	9.79e-25	89.4
APBKEJPL_01925	MER0001236	PepA aminopeptidase (*Escherichia coli*)	M17	34.8	97.8	3.50e-85	267
APBKEJPL_01880	MER0001482	family M20 unassigned peptidases (*Streptomyces coelicolor*)	NA	34.7	96.4	1.37e-55	184
APBKEJPL_02373	MER0001668	collagenase ({*Helicobacter*}-type) (*Clostridium beijerinckii*)	U32	34	92.2	1.21e-55	181
APBKEJPL_01887	MER0001793	family M38 unassigned peptidases (*Bacillus caldolyticus*)	NA	33.5	71.6	5.47e-31	117

APBKEJPL_00884	MER0000458	penicillin-binding protein 2 (*Bacillus subtilis*)	X52	33.4	52.9	6.27e-52	180
APBKEJPL_02692	MER0001582	subfamily M20A unassigned peptidases (*Nocardiopsis dassonvillei*)	NA	33.1	93.5	1.18e-59	196
APBKEJPL_00899	MER0001395	peptidase activation protein (*Lactococcus lactis*)	X15	31.3	94.7	3.82e-30	110
APBKEJPL_01317	MER0000352	lactocepin I (*Lactococcus lactis*)	S08	30.7	53.5	1.50e-33	129
APBKEJPL_02520	MER0000599	signalase (animal) 21 kDa component (*Canis familiaris*)	S26	30.7	65.5	2.18e-10	52.4
APBKEJPL_03058	MER0002013	At3g02875 ({*Arabidopsis thaliana*})-type peptidase (*Arabidopsis thaliana*)	NA	30.1	97.9	9.02e-44	152

Screening was performed by BLASTp of the predicted proteome against the MEROPS peptidase database, using a minimum identity of 30%, query coverage of 50%, and an E-value threshold of 1x10^{-5}. For each locus, only the best-matching MEROPS entry (highest bit score) is shown.

Table S2. Genes associated with heavy metal resistance and homeostasis identified in the genome of strain DLB3

DLB3 locus tag	BacMet gene	Associated metal(s)	Reference protein (BacMet)	Identity (%)	Query coverage (%)	E-value	Bit score
APBKEJPL_03302	yfmO	Cu	O06473	96.5	100.5	0	739
APBKEJPL_03303	yfmP	Cu	O06474	90	100	1.32e-89	252
APBKEJPL_01773	copZ	Cu	O32221	83.6	98.5	4.14e-37	114
APBKEJPL_00940	cueR	Cu	O07586	82.4	100.7	1.45e-82	234
APBKEJPL_01772	csoR	Cu	O32222	82.2	100	9.38e-60	173
APBKEJPL_01774	copA	Cu	O32220	78.9	99.4	0	1288
APBKEJPL_02971	ycnJ	Cu	C0SP95	67.7	100	0	721
APBKEJPL_02970	ycnK	Cu	P94433	66.1	99.5	1.52e-101	286
APBKEJPL_02331	ruvB	Cr; Te; Se	Q51426	61.7	99.4	6.49e-149	418
APBKEJPL_03578	mntH/yfeP	Mn; Fe; Cd; Co; Zn	P0A769	51.8	92.9	1.73e-133	385
APBKEJPL_01775	ziaA	Zn	Q59998	51.1	101	0	722
APBKEJPL_03323	oscA	Cr	B6CM35	46.5	75.4	4.23e-11	47.8
APBKEJPL_02621	nmtR	Ni; Cd; Pb	O69711	45.5	90	2.04e-24	85.1
APBKEJPL_03484	czcD	Cd; Zn; Co	P13512	44.8	92.7	7.78e-76	230
APBKEJPL_01994	chrR	Cr; Fe	Q7BD45	43.5	97.9	4.11e-24	85.5
APBKEJPL_02199	irlR	Cd; Zn	O31395	41.7	96.2	6.57e-48	153
APBKEJPL_02875	baeR	Zn; W	P69228	41.1	98.3	7.36e-54	169
APBKEJPL_01131	corR	Cu	Q1D6V8	40.7	70.9	6.72e-75	238
APBKEJPL_01124	pcoR	Cu	Q47456	39.3	87.1	6.74e-06	36.6
APBKEJPL_00267	recG	Cr; Te; Se	Q9HTL3	38.8	99	1.27e-136	412
APBKEJPL_03509	ricR	Cu	O07434	38.6	81.4	5.09e-11	49.3
APBKEJPL_01639	czcR	Cd; Zn; Co	Q44006	38.4	97.3	8.25e-44	142
APBKEJPL_01412	zraR/hydH	Zn	P14375	37.6	94.4	4.55e-19	76.6
APBKEJPL_01747	copR	Cu	Q02540	36	85.9	1.43e-32	113
APBKEJPL_03472	hmrR	Cu	Q9X5V4	36	82.8	2.11e-12	55.5
APBKEJPL_03071	znuC/yebM	Zn	P0A9X1	35.1	91.3	1.76e-33	116
APBKEJPL_02409	soxS	Zn	P0A9E2	31.4	80.8	1.63e-17	68.2

APBKEJPL_01640	irlS	Cd; Zn	O31396	31	73.9	2.10e-27	107
APBKEJPL_01507	chrA	Cr	P14285	30.7	98.9	2.80e-13	62
APBKEJPL_02110	troB	Zn; Mn; Fe	P96117	30.7	96.2	2.13e-23	90.5
APBKEJPL_02146	fieF/yiip	Fe; Zn; Co; Cd; Ni	P69380	30.5	94.1	6.35e-47	154
APBKEJPL_03070	znuB/yebI	Zn	P39832	30.1	93.5	2.78e-26	99.4
APBKEJPL_01268	arsB	As; Sb	P30329	67.2	98.8	0	577
APBKEJPL_02483	sodA	Se	P00448	57.7	103.5	4.93e-84	243
APBKEJPL_03067	terD	Te	P18781	57.4	98.4	2.23e-80	233
APBKEJPL_02489	pstB	As	P0AAH0	56.2	92.9	3.27e-107	306
APBKEJPL_03066	terE	Te	P18782	54.2	100	6.20e-76	221
APBKEJPL_00062	acn	Fe	O53166	51.6	103.9	0	933
APBKEJPL_00470	corT/coaT	Co	H0P0Y3	45.9	98.1	0	541
APBKEJPL_01802	fecE	Ni; Co	P15031	45.9	94.4	1.68e-84	248
APBKEJPL_01643	arsT	As	B7FB00	45.8	97.2	3.91e-85	254
APBKEJPL_01791	modB	W; Mo	P0AF01	42	79.7	3.37e-39	130
APBKEJPL_01014	nikD	Ni	P33593	40.6	71.3	7.21e-50	162
APBKEJPL_01801	fecD	Ni; Co	P15029	40.3	93.2	7.14e-62	195
APBKEJPL_00768	nikE	Ni	P33594	40.1	76.1	8.28e-52	167
APBKEJPL_01132	arsR	As; Sb; Bi	Q01256	39.7	71.6	6.92e-15	60.1
APBKEJPL_01010	wtpC	W; Mo	Q8U4K3	39.7	74.1	3.78e-36	126
APBKEJPL_01792	modA	W; Mo	Q72FN6	39.1	84.9	6.17e-53	168
APBKEJPL_00619	fetB/ybbM	Fe	P77307	38.8	95.7	6.57e-55	172
APBKEJPL_02531	mntR	Mn; Mg	P0A9F1	37.7	74.6	2.93e-20	76.6
APBKEJPL_00775	nikC	Ni	P0AFA9	37.6	80.6	7.81e-51	164
APBKEJPL_00965	glpF	Sb; As	P0AER0	37	89.5	1.03e-28	105
APBKEJPL_02996	modC	W; Mo	Q72FN3	36.6	76.7	2.12e-32	114
APBKEJPL_02054	dpr/dps	Fe	P0CB53	36	95.9	1.02e-29	101
APBKEJPL_00776	nikB	Ni	P33591	36	101.9	1.15e-64	202
APBKEJPL_02086	fbpC	Fe; Ga	P44513	35.8	88.3	5.10e-36	125
APBKEJPL_03189	ybtP	Fe	Q9R7V3	35.1	80.2	2.95e-78	254
APBKEJPL_00387	merA	Hg	P0A0E5	34.9	96.4	4.29e-87	272

APBKEJPL_00110	merR2	Hg	Q9WWL1	34.6	77.6	4.40e-07	41.2
APBKEJPL_01300	frnE	Cd	Q9RWK7	34	103	1.14e-26	97.8
APBKEJPL_02088	pmrA	Fe	Q70FH0	33.5	77.5	3.05e-16	70.1
APBKEJPL_01915	yfeB	Fe; Mn	Q56953	31.8	85.9	9.30e-24	92
APBKEJPL_03225	ybtQ	Fe	Q9Z375	31.7	83	5.36e-70	233
APBKEJPL_01833	dpsA	Fe	Q8KR86	31.4	99.4	7.71e-25	89.4
APBKEJPL_03159	cnrH	Co; Ni	P37978	31	93	4.90e-17	70.5
APBKEJPL_03053	emrB	NA	P0AEJ0	31	87.3	6.02e-54	185
APBKEJPL_03679	klaB/telA/kilB	Te	Q52328	30.7	94.6	9.39e-49	164
APBKEJPL_01432	arsC	As	Q01257	30.6	88.7	4.87e-13	57.8

Screening was performed by BLASTp (v2.17.0) against the BacMet experimentally confirmed database (v2.0), using a minimum amino-acid identity of 30%, query coverage of 70%, and an E-value threshold of $1x10^{-5}$. For each BacMet reference gene, only the best-matching DLB3 protein (highest bit score) is shown; metal associations follow BacMet database annotation and may include multiple metals per gene.

Table S3. Antibiotic resistance genes identified in the genome of strain DLB3

DLB3 locus tag	Best hit (ARO)	AMR gene family	Drug class	Resistance mechanism	Identity (%)	Reference length (%)	Bit score	Associated antibiotic
APBKEJPL_03477	clbA	Cfr 23S ribosomal RNA methyltransferase	lincosamide antibiotic; streptogramin antibiotic; streptogramin A antibiotic; oxazolidinone antibiotic; phenicol antibiotic; pleuromutilin antibiotic	antibiotic target alteration	97.4	100	685.6	lincomycin; clindamycin; azidamfenicol; chloramphenicol; thiamphenicol; virginiamycin M1; tiamulin; madumycin II; griseoviridin; dalfopristin
APBKEJPL_02679	vanY gene in vanB cluster	vanY; glycopeptide resistance gene cluster	glycopeptide antibiotic	antibiotic target alteration	34.9	101.5	105.1	vancomycin
APBKEJPL_03547	vanT gene in vanG cluster	glycopeptide resistance gene cluster; vanT	glycopeptide antibiotic	antibiotic target alteration	34.8	54.6	197.2	vancomycin
APBKEJPL_02561	vanT gene in vanG cluster	glycopeptide resistance gene cluster; vanT	glycopeptide antibiotic	antibiotic target alteration	33.7	55.2	201.1	vancomycin
APBKEJPL_02405	tet(45)	major facilitator superfamily (MFS) antibiotic efflux pump	tetracycline antibiotic	antibiotic efflux	75.3	100	691	tetracycline
APBKEJPL_00644	BcI	class A Bacillus cereus Bc beta-lactamase	cephalosporin	antibiotic inactivation	63.1	99.3	389.8	-

APBKEJPL_00799	FosBx1	fosfomycin thiol transferase	phosphonic acid antibiotic	antibiotic inactivation	62.8	101.5	191.8	fosfomycin
APBKEJPL_01068	qacJ	small multidrug resistance (SMR) antibiotic efflux pump	disinfecting agents and antiseptics	antibiotic efflux	55.1	118.7	124.8	benzalkonium chloride
APBKEJPL_00126	qacJ	small multidrug resistance (SMR) antibiotic efflux pump	disinfecting agents and antiseptics	antibiotic efflux	44.9	109.3	92	benzalkonium chloride
APBKEJPL_00125	qacJ	small multidrug resistance (SMR) antibiotic efflux pump	disinfecting agents and antiseptics	antibiotic efflux	37.4	102.8	83.6	benzalkonium chloride

Table S4. Pan-genome composition of strain DLB3 and nine reference genomes (eight *B. velezensis* strains and *B. amyloliquefaciens* DSM7)

Gene category	Definition (% of strains carrying the gene)	No. of gene clusters	% of pan-genome
Core genes	99% ≤ strains ≤ 100%	2486	35.7%
Soft core genes	95% ≤ strains < 99%	0	0.0%
Shell genes	15% ≤ strains < 95%	2294	33.0%
Cloud genes	0% ≤ strains < 15%	2179	31.3%
**Total (pan-genome)**	**0% ≤ strains ≤ 100%**	**6959**	**100.0%**

Table S5. DLB3-specific gene clusters identified by Roary pan-genome analysis.

DLB3 locus tag	Gene name	Predicted annotation (Prokka)
APBKEJPL_00020	bioI	Biotin biosynthesis cytochrome P450
APBKEJPL_00036		hypothetical protein
APBKEJPL_00085		hypothetical protein
APBKEJPL_00087		hypothetical protein
APBKEJPL_00091		hypothetical protein
APBKEJPL_00094		hypothetical protein
APBKEJPL_00095		hypothetical protein
APBKEJPL_00098		hypothetical protein
APBKEJPL_00103		hypothetical protein
APBKEJPL_00124		hypothetical protein
APBKEJPL_00171		putative MFS-type transporter YhhS
APBKEJPL_00290	sirC	Precorrin-2 dehydrogenase
APBKEJPL_00529		hypothetical protein
APBKEJPL_00531		hypothetical protein
APBKEJPL_00532		hypothetical protein
APBKEJPL_00556		hypothetical protein
APBKEJPL_00558		hypothetical protein
APBKEJPL_00602		hypothetical protein
APBKEJPL_00617		hypothetical protein
APBKEJPL_00629		hypothetical protein
APBKEJPL_00631		hypothetical protein
APBKEJPL_00645	yjcQ	putative protein YjcQ
APBKEJPL_00646		hypothetical protein
APBKEJPL_00647		hypothetical protein
APBKEJPL_00648		hypothetical protein
APBKEJPL_00649		hypothetical protein
APBKEJPL_00650		hypothetical protein
APBKEJPL_00651		hypothetical protein

APBKEJPL_00652	hypothetical protein
APBKEJPL_00653	hypothetical protein
APBKEJPL_00656	hypothetical protein
APBKEJPL_00657	hypothetical protein
APBKEJPL_00658	hypothetical protein
APBKEJPL_00659	hypothetical protein
APBKEJPL_00660	hypothetical protein
APBKEJPL_00661	hypothetical protein
APBKEJPL_00662	hypothetical protein
APBKEJPL_00663	hypothetical protein
APBKEJPL_00665	hypothetical protein
APBKEJPL_00666	hypothetical protein
APBKEJPL_00667	hypothetical protein
APBKEJPL_00668	hypothetical protein
APBKEJPL_00669	hypothetical protein
APBKEJPL_00671	hypothetical protein
APBKEJPL_00673	hypothetical protein
APBKEJPL_00677	hypothetical protein
APBKEJPL_00678	hypothetical protein
APBKEJPL_00679	hypothetical protein
APBKEJPL_00680	hypothetical protein
APBKEJPL_00681	hypothetical protein
APBKEJPL_00683	hypothetical protein
APBKEJPL_00684	hypothetical protein
APBKEJPL_00685	hypothetical protein
APBKEJPL_00686	hypothetical protein
APBKEJPL_00687	hypothetical protein
APBKEJPL_00688	hypothetical protein
APBKEJPL_00689	hypothetical protein
APBKEJPL_00690	hypothetical protein
APBKEJPL_00694	hypothetical protein

APBKEJPL_00695		hypothetical protein
APBKEJPL_00696		hypothetical protein
APBKEJPL_00697		hypothetical protein
APBKEJPL_00699		hypothetical protein
APBKEJPL_00700		hypothetical protein
APBKEJPL_00701		hypothetical protein
APBKEJPL_00702	dnaA_2	Chromosomal replication initiator protein DnaA
APBKEJPL_00703		hypothetical protein
APBKEJPL_00704		hypothetical protein
APBKEJPL_00705		hypothetical protein
APBKEJPL_00706		hypothetical protein
APBKEJPL_00707		hypothetical protein
APBKEJPL_00708	yqaI	putative protein YqaI
APBKEJPL_00711		hypothetical protein
APBKEJPL_00712		hypothetical protein
APBKEJPL_00713		HTH-type transcriptional activator BcrR
APBKEJPL_00714	xre_2	HTH-type transcriptional regulator Xre
APBKEJPL_00715		hypothetical protein
APBKEJPL_00716		hypothetical protein
APBKEJPL_00717	xerC_2	Tyrosine recombinase XerC
APBKEJPL_00724		hypothetical protein
APBKEJPL_00732		hypothetical protein
APBKEJPL_00816		hypothetical protein
APBKEJPL_00826		hypothetical protein
APBKEJPL_00845		hypothetical protein
APBKEJPL_00876		hypothetical protein
APBKEJPL_00880		hypothetical protein
APBKEJPL_00978		hypothetical protein
APBKEJPL_01022	pepE	Peptidase E
APBKEJPL_01023		hypothetical protein
APBKEJPL_01024		hypothetical protein

APBKEJPL_01068		Quaternary ammonium compound-resistance protein QacC
APBKEJPL_01069	immR	HTH-type transcriptional regulator ImmR
APBKEJPL_01082		hypothetical protein
APBKEJPL_01146		hypothetical protein
APBKEJPL_01147		hypothetical protein
APBKEJPL_01148		Iron-sulfur cluster carrier protein
APBKEJPL_01151	pucA	putative xanthine dehydrogenase subunit A
APBKEJPL_01152	pucB	Purine catabolism protein PucB
APBKEJPL_01162	rep	ATP-dependent DNA helicase Rep
APBKEJPL_01213		RecBCD enzyme subunit RecD
APBKEJPL_01220		hypothetical protein
APBKEJPL_01221		hypothetical protein
APBKEJPL_01222		hypothetical protein
APBKEJPL_01223		hypothetical protein
APBKEJPL_01224		Immunity protein WapI
APBKEJPL_01225		hypothetical protein
APBKEJPL_01226		hypothetical protein
APBKEJPL_01228		hypothetical protein
APBKEJPL_01232	licT	Transcription antiterminator LicT
APBKEJPL_01233		hypothetical protein
APBKEJPL_01240	rlmA	23S rRNA (guanine(745)-N(1))-methyltransferase
APBKEJPL_01313	ysnE	putative N-acetyltransferase YsnE
APBKEJPL_01339	murG_2	UDP-N-acetylglucosamine--N-acetylmuramyl-(pentapeptide) pyrophosphoryl-undecaprenol N-acetylglucosamine transferase
APBKEJPL_01390		hypothetical protein
APBKEJPL_01391	rapF_1	Response regulator aspartate phosphatase F
APBKEJPL_01451		hypothetical protein
APBKEJPL_01503		hypothetical protein
APBKEJPL_01605		IS256 family transposase ISTsu1
APBKEJPL_01606		hypothetical protein
APBKEJPL_01607		Putative transposase InsK for insertion sequence element IS150

APBKEJPL_01638		hypothetical protein
APBKEJPL_01656		hypothetical protein
APBKEJPL_01668		hypothetical protein
APBKEJPL_01724	rfbE	GDP-perosamine synthase
APBKEJPL_01746		hypothetical protein
APBKEJPL_01758		Barstar
APBKEJPL_01846	bmr3_2	Multidrug resistance protein 3
APBKEJPL_01883		hypothetical protein
APBKEJPL_01884		hypothetical protein
APBKEJPL_01885		hypothetical protein
APBKEJPL_01958		hypothetical protein
APBKEJPL_01959		Competence pheromone
APBKEJPL_01960	comP	Sensor histidine kinase ComP
APBKEJPL_01970		hypothetical protein
APBKEJPL_01996		hypothetical protein
APBKEJPL_02078	pksJ_2	Polyketide synthase PksJ
APBKEJPL_02251		hypothetical protein
APBKEJPL_02252		hypothetical protein
APBKEJPL_02298		hypothetical protein
APBKEJPL_02328		hypothetical protein
APBKEJPL_02611		hypothetical protein
APBKEJPL_02612		hypothetical protein
APBKEJPL_02687		hypothetical protein
APBKEJPL_02691	yodP	N-acetyltransferase YodP
APBKEJPL_02708		hypothetical protein
APBKEJPL_02711		hypothetical protein
APBKEJPL_02713		hypothetical protein
APBKEJPL_02718		hypothetical protein
APBKEJPL_02724		hypothetical protein
APBKEJPL_02726	yobL_1	Ribonuclease YobL
APBKEJPL_02727		hypothetical protein

APBKEJPL_02737		hypothetical protein
APBKEJPL_02771		hypothetical protein
APBKEJPL_02939		hypothetical protein
APBKEJPL_03027	bglC	Aryl-phospho-beta-D-glucosidase BglC
APBKEJPL_03046		hypothetical protein
APBKEJPL_03128		Mitomycin biosynthesis 6-O-methyltransferase
APBKEJPL_03129	dltA_4	D-alanine--D-alanyl carrier protein ligase
APBKEJPL_03130		1-hydroxycarotenoid 3,4-desaturase
APBKEJPL_03131		Polyketide synthase PksN
APBKEJPL_03132		Polyketide synthase PksN
APBKEJPL_03133		Malonyl CoA-acyl carrier protein transacylase
APBKEJPL_03134		Linear gramicidin synthase subunit D
APBKEJPL_03135		Tyrocidine synthase 2
APBKEJPL_03136		Linear gramicidin dehydrogenase LgrE
APBKEJPL_03137		hypothetical protein
APBKEJPL_03138	lnrN_2	Linearmycin resistance permease protein LnrN
APBKEJPL_03139	lnrM	Linearmycin resistance permease protein LnrM
APBKEJPL_03140	lnrL_4	Linearmycin resistance ATP-binding protein LnrL
APBKEJPL_03141		Transcriptional regulatory protein LnrK
APBKEJPL_03142		hypothetical protein
APBKEJPL_03149		Ubiquinone biosynthesis O-methyltransferase, mitochondrial
APBKEJPL_03150		hypothetical protein
APBKEJPL_03151	ramC	putative SapB synthase
APBKEJPL_03176	ybbA	putative protein YbbA
APBKEJPL_03194	yfhS	putative protein YfhS
APBKEJPL_03238		hypothetical protein
APBKEJPL_03293		hypothetical protein
APBKEJPL_03299		hypothetical protein
APBKEJPL_03334		hypothetical protein
APBKEJPL_03336		hypothetical protein
APBKEJPL_03338	yezG_1	putative antitoxin YezG

APBKEJPL_03339	yezG_2	putative antitoxin YezG
APBKEJPL_03465		hypothetical protein
APBKEJPL_03474		putative formaldehyde dehydrogenase AdhA
APBKEJPL_03483	czcO	putative oxidoreductase CzcO
APBKEJPL_03485		hypothetical protein
APBKEJPL_03487		hypothetical protein
APBKEJPL_03489	mutT	8-oxo-dGTP diphosphatase
APBKEJPL_03502		hypothetical protein
APBKEJPL_03503	racX	Broad specificity amino-acid racemase RacX
APBKEJPL_03518	yobL_2	Ribonuclease YobL
APBKEJPL_03519	yobK_2	Antitoxin YobK
APBKEJPL_03520		hypothetical protein
APBKEJPL_03521		hypothetical protein
APBKEJPL_03522		hypothetical protein
APBKEJPL_03524	rapI_3	Response regulator aspartate phosphatase I
APBKEJPL_03525		hypothetical protein
APBKEJPL_03526		hypothetical protein
APBKEJPL_03570		hypothetical protein
APBKEJPL_03582		hypothetical protein
APBKEJPL_03585	rppH	RNA pyrophosphohydrolase

Table S6. Distribution and similarity confidence of biosynthetic gene clusters in DLB3, eight *B. velezensis* strains and *B. amyloliquefaciens* DSM7 with their most similar known clusters

Cluster	DLB3	FZB42	CBMB205	KACC 18228	KACC 13105	NRRL B-41580	12Y	10075	HNI10	DSM 7
Fengycin	High	High	High	High	High	High	High	High	High	High
Bacillaene	High	High	High	High	High	High	Medium	High	High	High
Macrolactin H	High	High	High	High	High	High	High	High	High	-
Bacilysin	High	High	High	High	High	High	High	High	High	High
Bacillibactin	High	High	High	High	High	High	High	High	High	High
Bacillothiazol A-N	High	High	-	-	-	-	-	-	-	-
Difficidin	Medium	High	High	Medium	High	High	High	High	High	-
Surfactin	High	High	High	High	Medium	Low	High	High	High	High
Mersacidin	-	-	-	-	-	High	-	-	-	-
Plipastatin	-	-	-	Low	Low	-	-	-	-	-
Plantazolicin	-	High	-	-	-	-	High	High	High	-
Andalusicin A/B	High	-	-	-	-	-	-	-	-	-

High, medium, and low indicate the confidence level of biosynthetic gene cluster prediction based on antiSMASH analysis. Only clusters with a "most similar known cluster" annotation were included in this comparison." –" : cluster absent.
